# The effectiveness of manual and exercise therapy on headache intensity and frequency among patients with cervicogenic headache: a systematic review and meta-analysis

**DOI:** 10.1186/s12998-022-00459-9

**Published:** 2022-11-23

**Authors:** Pietro Bini, David Hohenschurz-Schmidt, Vincenzo Masullo, Diana Pitt, Jerry Draper-Rodi

**Affiliations:** 1 Department of Surgery and Cancer, Faculty of Medicine, Imperial College, London, Chelsea and Westminster Hospital, 4Th Floor, 369 Fulham Road, London, SW10 9NH UK; 2 University College of Osteopathy, 275 Borough High Street, London, SE1 1JE UK; 3 Department of Applied Social Science and Social Practice, Ara Institute of Canterbury, Madras Campus, “O” Building, Madras street, Christchurch Central City, Christchurch, 8011 New Zealand

**Keywords:** Exercise therapy, Meta-analysis, Musculoskeletal manipulation, Post-traumatic headache, Randomized controlled trial, Systematic review

## Abstract

**Background:**

Cervicogenic headache is a secondary headache, and manual therapy is one of the most common treatment choices for this and other types of headache. Nonetheless, recent guidelines on the management of cervicogenic headache underlined the lack of trials comparing manual and exercise therapy to sham or no-treatment controls. The main objective of this systematic review and meta-analysis was to assess the effectiveness of different forms of manual and exercise therapy in people living with cervicogenic headache, when compared to other treatments, sham, or no treatment controls.

**Methods:**

Following the PRISMA guidelines, the literature search was conducted until January 2022 on MEDLINE, CENTRAL, DOAJ, and PEDro. Randomized controlled trials assessing the effects of manual or exercise therapy on patients with cervicogenic headache with headache intensity or frequency as primary outcome measures were included. Study selection, data extraction and Risk of Bias (RoB) assessment were done in duplicate. GRADE was used to assess the quality of the evidence.

**Results:**

Twenty studies were included in the review, with a total of 1439 patients. Common interventions were spinal manipulation, trigger point therapy, spinal mobilization, scapulo-thoracic and cranio-cervical exercises. Meta-analysis was only possible for six manual therapy trials with sham comparators. Data pooling showed moderate-to-large effects in favour of manual therapy for headache frequency and intensity at short-term, small-to-moderate for disability at short-term, small-to-moderate for headache intensity and small for headache frequency at long-term. A sensitivity meta-analysis of low-RoB trials showed small effects in favor of manual therapy in reducing headache intensity, frequency and disability at short and long-term. Both trials included in the sensitivity meta-analysis studied spinal manipulation as the intervention of interest. GRADE assessment showed moderate quality of evidence.

**Conclusion:**

The evidence suggests that manual and exercise therapy may reduce headache intensity, frequency and disability at short and long-term in people living with cervicogenic headache, but the overall RoB in most included trials was high. However, a sensitivity meta-analysis on low-RoB trials showed moderate-quality evidence supporting the use of spinal manipulation compared to sham interventions. More high-quality trials are necessary to make stronger recommendations, ideally based on methodological recommendations that enhance comparability between studies.

*Trial registration* The protocol for this meta-analysis was pre-registered on PROSPERO under the registration number CRD42021249277.

## Introduction

### Background

**Fig. 1:**
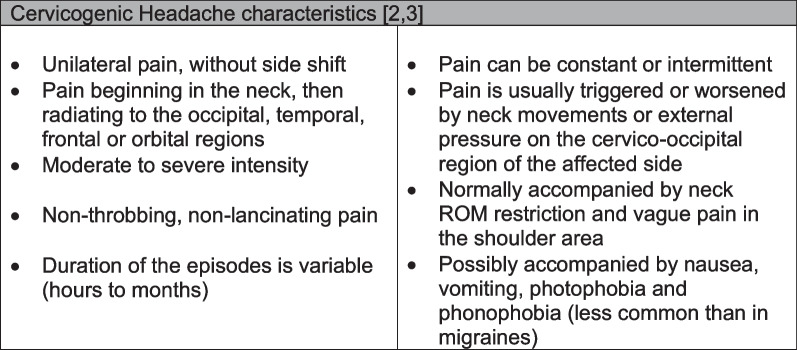
Common CGH features reported by the International Headache Society [2] and the Cervicogenic Headache International Study Group [3] are described

Cervicogenic Headache (CGH) is a secondary headache, with a prevalence of 1–4% among people experiencing headaches [1]. The pathophysiological mechanism underlying this condition is referred pain, and the currently accepted theory is that structures in the upper cervical spine supplied by the first three spinal nerves can refer pain to the occipital, frontal or temporal regions. Specific features which tend to characterize CGH and are considered in the diagnostic process are presented in Fig. 1 [1, 2]. Several sets of criteria have been proposed. Most widely used are the criteria proposed by the International Headache Society (IHS) in the International Classification of Headache Disorders-3rd version (ICHD) [2], and the ones proposed by the Cervicogenic Headache International Study Group (CHISG) [3]. Despite the presence of features characteristic of CGH, different headaches with similar phenotypes can co-exist, posing further obstacles to the diagnostic process [4].

Manual therapy is among the most common treatment choices for headaches in Australia, Europe and in the USA, provided to about a third of patients in headache clinics [5]. Recent guidelines on the management of CGH [6] support the use of exercise therapy and spinal manipulation to reduce CGH pain intensity, frequency, and disability. Based on the current literature, an initial 8–10 sessions of manual or exercise therapy (i.e. low-load endurance exercise, spinal manipulation or mobilization) over 6 weeks are recommended in isolation [6].

While this recommendation is supported by three trials, the guideline authors highlight the lack of high-quality studies studying the efficacy of non-pharmacological interventions compared to sham or no treatment [6]. Two systematic reviews and meta-analyses [7, 8] in this field evaluated the effectiveness of spinal manipulation (alone or combined with mobilization) for CGH and tension-type headache. One systematic review [7] found no evidence in favour of spinal manipulation compared to other conservative interventions for headache intensity or disability. This review compared manipulation and mobilization to other forms of manual therapy and various forms of exercise, but it did not assess the effectiveness of other interventions commonly used by manual therapists (i.e. massage, exercise, and acupuncture), nor the efficacy of manual therapy compared to no treatment or sham. The second systematic review [8] did include sham-controlled randomized controlled trials (RCTs), but grouped sham interventions with “other forms of manual therapy''. RCTs using sham and other manual therapy interventions as comparisons were incorporated into the same meta-analysis, not allowing for a separate appraisal of RCTs with sham-controls only. The review reported spinal manipulation as more effective in the short-term for headache intensity, frequency and disability, and in the medium-term for headache frequency, but it did not allow for conclusions on comparisons to sham controls.

Our systematic review has a broader scope than the previous reviews, comparing the effectiveness of interventions commonly used in a manual therapy setting to other conservative interventions, as well as to sham or no treatment. This allows for the assessment of intervention efficacy against control interventions that ideally account for expectancy effects, rather than basing recommendations solely on comparative effectiveness studies, which can lead to bias [9]. Such recommendations would help to inform patients and clinicians on the appropriateness of choosing manual and exercise therapy for the management of CGH in general, and against or alongside other possible treatment options.

### Objectives

The objective of this systematic review was to systematically review the effectiveness and efficacy of manual and exercise therapy for CGH intensity and frequency when compared to placebo, no treatment or other interventions.

## Methods

The systematic review was performed following the PRISMA guidelines [10].

### Literature search

**Fig. 2:**
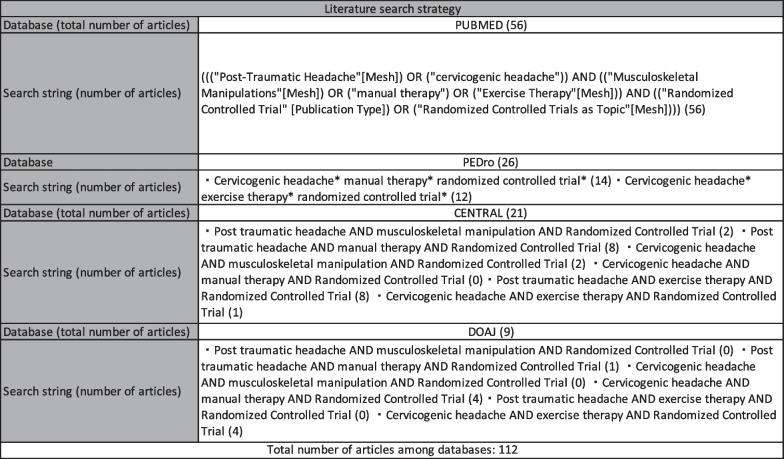
The search strategy for Pubmed, PEDro, CENTRAL, and DOAJ with the related number of articles retrieved are reported

A computerized search was conducted for the following electronic databases: MEDLINE (via PubMed), CENTRAL, DOAJ, and PEDro. EMBASE, MEDNAR and SAGE databases were not consulted due to access limitation, deviating from the protocol of this systematic review. The search was from inception to December 2020, and was updated in January 2022 to include trials published after December 2020. No language restrictions were applied during the search, but studies were excluded if English, German, Italian, Spanish or Portuguese language versions were not available in the literature. References of the included studies were searched manually, and content experts consulted to ensure that no relevant literature was missed. The complete search strategy is provided in Fig. 2.

### Eligibility criteria

The research question and eligibility criteria were designed following the PICOS (Participant, Intervention, Comparator, Outcomes, Study design) method [11].

#### Participants

Trials with people diagnosed with CGH according to the IHS [2] or the CHISG [3] diagnostic criteria were included, regardless of participants’ age, gender or symptoms duration. Trials using modified versions of the aforementioned criteria were also included, in line with previous Cochrane reviews [12]. Nonetheless, for transparency reasons and to allow potential secondary analyses on the impact of diagnostic criteria on trial results, studies that did not state the diagnostic criteria used for CGH were excluded.

#### Intervention(s)

Manual therapy is defined as any techniques administered manually by a trained practitioner for therapeutic purposes [13]. For the scope of this systematic review, manual therapy techniques of interest included massage, trigger point therapy, kinesio-taping, manipulation, mobilization, acupuncture (including dry needling) or a combination of such techniques. Exercise therapy involves movement prescribed to correct impairments, restore muscular and skeletal function, and/or maintain a state of well-being. Therapeutic exercise modalities considered for inclusion by this systematic review were: endurance training (i.e. low-load endurance exercises), resistance training (isotonic, isometric and isokinetic exercises), flexibility training (static and dynamic mobility exercises, stretching exercises) [14]. Studies which included a combination of manual and exercise therapy interventions (i.e. spinal manipulation and stretching exercises; trigger point therapy and low-load endurance exercises) were included. Trials using reflexology, acupressure, wellness massage or Reiki as the experimental intervention of interest were excluded.

#### Comparator(s)/control conditions

Eligible comparators were sham and placebo controls, no treatment, and other active interventions.

#### Main outcome(s)

Headache intensity, disability, frequency and duration are commonly used outcomes for headaches [15, 16] and have been considered by previous guidelines [6]. The primary outcome measures of interest were headache intensity and frequency, and trials not including these outcome measures were considered ineligible for the systematic review. Secondary outcomes of interest were disability and headache duration.

#### Study design

Only prospective randomized controlled trials were included. Case reports and case series, observational studies, and crossover studies were not eligible.

### Screening and eligibility assessment

The literature search and de-duplication were carried out by a single researcher (PB). Search results were imported to Endnote^©^ and duplicates removed using the Endnote tool. Subsequent screening performed in Covidence [17], an online platform for systematic reviews.

The study selection was performed in duplicate by two independent reviewers (PB and VM), initially based on study titles and abstracts, and followed by full-text screening, using a pre-defined study eligibility form on an offline spreadsheet in conjunction with Covidence, where decisions on inclusion/exclusion of trials were made. Disagreements were discussed by the two reviewers, and mediated by a third party if necessary. Screening procedures were pre-tested by calculating a Kappa score on a sub-sample of retrieved studies [18].

### Data extraction

Two independent reviewers (PB and VM) extracted data using a predefined data extraction form on an offline spreadsheet, and consensus was reached by discussion and mediation in case of disagreements. Data were extract for: author and date of the trial, experimental and control interventions studied, primary and secondary outcome measures considered, duration and frequency of the intervention, and follow-up measurements times. For the primary and secondary outcome measures of interest for this review, values were extracted for all reported time points and groups. Where necessary, data were extracted from figures using the Adobe Reader^©^ measurement tool.

A description of potentially relevant studies excluded at the full-text screening stage with reasons for exclusion was provided in the results section.

### Risk of bias assessment

Risk of bias (RoB) assessments were performed by two reviewers (PB and VM) using the criteria proposed by the Cochrane Back and Neck Group [19], and consensus was reached by discussion when needed. Inter-rater reliability was assessed using Kappa score [18]. As recommended by the authors of the RoB tool, trials were not categorized according to arbitrary cut-off points of the overall score. Instead, studies were considered as overall low-RoB if no individual domain was rated as “high” or “unsure” RoB. Studies which scored “unsure”, but not “high”, for one or more items, were considered as overall unsure RoB, and “high” if any individual item was rated as high RoB.

A common concern in manual and exercise therapy studies is the lack of blinding of patients, providers, or both [20, 21]. Obscuring treatment allocation from patients, and in particular therapists, is inherently difficult due to the complex and participatory nature of most interventions [22]. To avoid unduly skewing RoB assessments, and aligning with a previous meta-analysis of physiotherapy for headaches [12], the items “patient blinding”, “assessor blinding” and “therapist blinding” were considered non-applicable. Following methodology recommendations, the RoB assessment for each trial was outcome-specific [23]. Considering that the primary and secondary outcome measures of interest of this systematic review constituted of subjective outcome measures, the RoB assessment for headache intensity, frequency, duration and disability could be summarized across these outcomes. Where objective or clinically-observed outcomes were evaluated, a separate RoB assessment was provided.

### Data synthesis

#### Descriptive analysis

A detailed description of study characteristics and RoB assessments was provided in the results section. For the descriptive analysis, trials were sub-grouped according to the specific experimental intervention used. Data from the included trials were presented in a summary table.

In order to determine whether statistically significant changes constituted important clinical benefits or detriments for patients, Minimal Clinical Important Differences (MCIDs) were analyzed when available from the literature for a specific outcome measure. The MCID is defined as the smallest difference in score in any outcome that patients can perceive as beneficial or harmful. MCIDs allow for the appreciation of patients’ perspectives on their health and treatments, making MCIDs an important factor in decision-making [24]. To facilitate the interpretation of the findings, RoB judgements and estimates of outcomes, as well as available data on statistical (P value) and clinical significance (MCIDs) were described in separate summary tables.

Regarding MCIDs for the outcome measures of interest in this systematic review, headache intensity is often assessed via a Visual Analogue Scale (VAS) or Numeric Pain Rating Scale (NPRS), headache frequency is often reported as “number of days with headache in last 2 or 4 weeks”, and disability as the Neck Disability Index (NDI). The aforementioned pain scales have been shown to be reliable in assessing pain intensity and disability [7, 25, 26, 28]. Nonetheless, MCIDs of these scales for CGH have only been derived for NPRS (2.5-point reduction after 4 weeks of intervention) [16], NDI (5.5-point reduction at 4 weeks) [16], and headache frequency (50% reduction of days with headache) [29]. MCIDs for headache duration were not found in the literature. Throughout the Results and Discussion, findings were only contextualized with MCIDs when these were available from the literature for the respective outcome measure.

#### Meta-analysis

The quantitative synthesis was performed using RevMan 5 (Review Manager 5 software, Version 5.4) [30]. For continuous outcomes, studies were compared using standardized mean differences (SMDs) and standard deviations (SDs). In cases of missing data, study authors were contacted. If the missing data were not accessible and not imputable from other reported data, articles were excluded from quantitative analyses. Q statistics and I^2^ were used to assess statistical heterogeneity. Random effects models were employed to calculate overall effects, and forest plots to depict estimates. SMDs between 0.2 and 0.5 were considered as small effect sizes, SMD between 0.5 and 0.8 moderate effect sizes, and SMD > 0.8 were considered large effect sizes [31].

Due to large differences in the designs of the included trials, the strategy for data pooling was changed from the one proposed in the protocol to allow for a more nuanced interpretation of the findings. Studies were compared only when the control interventions were comparable (i.e. grouping trials with sham or placebo controls, trials with no-treatment controls, and trials with other interventions), and pooling was divided into short-term (< 3 months) and long-term (> 3 months) endpoints, in line with previous systematic reviews on this topic [32]. When a single study reported multiple outcome assessments within the same time period (e.g. 2 or more follow-ups before 3 months), data for the time point closest to the other pooled studies were used. When trials with high or unsure RoB were included in the meta-analysis, a sensitivity analysis was also conducted, excluding the high or unsure RoB studies.

#### GRADE assessment

The GRADE (Grading of Recommendations Assessment, Development and Evaluation) approach [33] was used to evaluate the overall quality of the evidence for each outcome of interest. In brief, the overall quality of evidence for each pooled estimate was initially considered “high”, and could be downgraded by 1 level for each of the following 5 criteria: RoB (any of the trials included in the analysis showed “high” or “unsure” RoB [34], inconsistency (large heterogeneity among trials, I^2^ > 50%) [35], imprecision (< 400 participants for each comparison) [36], indirectness (indirectness of population, outcomes or intervention) [37], and publication bias (which was assessed with a funnel plot and Egger’s test if 10 or more studies were pooled) [38]. Two reviewers (PB and VM) applied the criteria. A GRADE profile was completed for each pooled estimate. The following definitions of quality of the evidence were applied [39]: high quality (further research is very unlikely to change our confidence in the estimate of effect), moderate quality (further research is likely to have an important effect on our confidence in the estimate of effect and may change the estimate), low quality (further research is very likely to have an important effect on our confidence in the estimate of effect and is likely to change the estimate), and very low quality (we are very uncertain about the estimate).

## Results

**Fig. 3:**
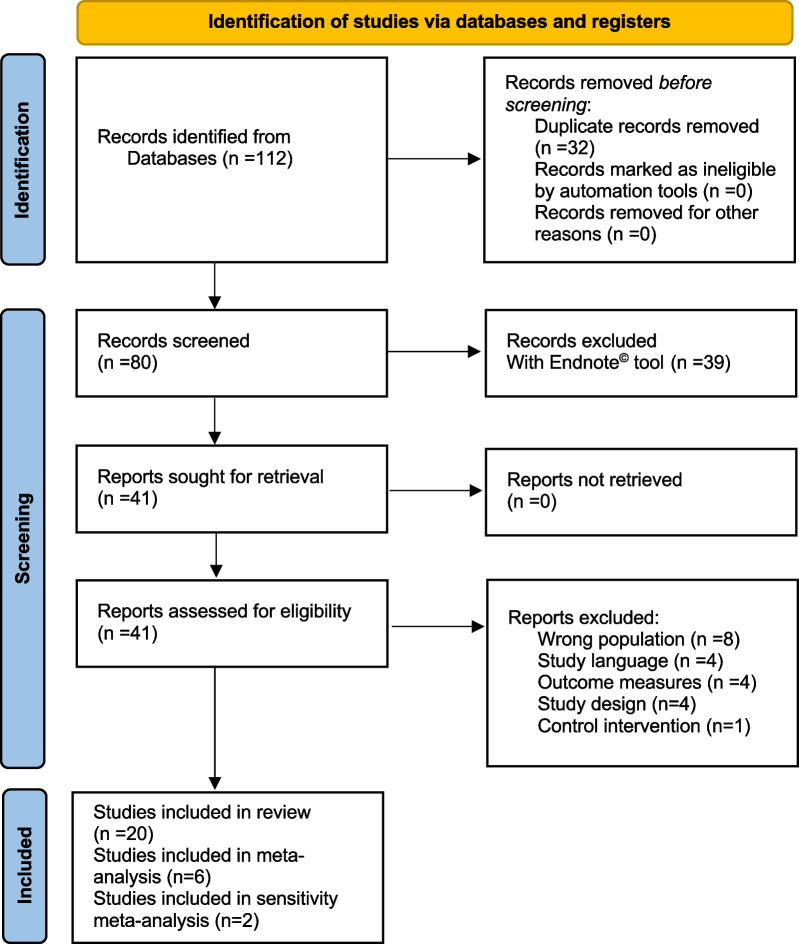
PRISMA flow diagram of the systematic search and selection process

The detailed process of study selection performed in January 2022 is presented in the PRISMA flow diagram (Fig. 3).

After deduplication, the literature search identified 80 potentially relevant trials. Twenty studies were included in the final review, with a total of 1439 patients. The eligibility assessment had strong inter-rater reliability (Cohen’s Kappa = 0.92).

Trials were mainly excluded during the full-text screening due to ineligible pathologies (i.e. different headaches) [40–44], outcome measures [45–48], and unclear diagnostic criteria for CGH [49, 50]. Table 1 provides the characteristics of the included trials.

**Table 1:** For each study, the type of intervention and comparators, the number of participants for each group, all outcome measures analyzed, the duration and frequency of the intervention and the time-points for each follow-up measurement are reported

Authors	Intervention (N)	Intervention description	Comparator (N)	Primary outcome measures	Secondary outcome measures	Duration and frequency of intervention	Measurement time points
Bodes-Pardo et al. [51]	Trigger point therapy + stretching (10)	Passive progressive manual pressure over trigger points (hyperirritable spot within a taut band of a skeletal muscle that elicits a referred pain upon examination)	Sham trigger point therapy (10)	HI (NPRS 0–10)	Cervical ROM (degrees). Pressure pain sensitivity (analogical algometer). Deep cervical flexors motor performance (CCFT)	3 sessions over 1 week	Baseline; 1 week
Chaibi et al. [52]	Cervical manipulation (4)	High-velocity, low-amplitude spinal manipulation	Sham manual therapy (4); Control (4)	HI (VAS 0–10), HF (days/month)	HD (hours/day), Headache Index (HI × HF × HD)	12 sessions over 3 months	Baseline; 3 months; 6 months; 12 months
Dunning et al. [53]	Cervical and thoracic manipulation (58)	High-velocity, low-amplitude spinal manipulation	Cervical and thoracic mobilization and cranio-cervical flexion exercises (52)	HI (NPRS 0–10), HF (days/week)	HD (hours/week), Disability (NDI), Global Rating of Change Scale (GRCS), Medictions intake	6–8 sessions over 4 weeks	Baseline; 1 week; 4 weeks; 3 months
Esin et al. [54]	Kinesiotaping (34)	Application of elastic tape over the vertical and horizontal portions of the trapezius muscle	Sham taping (34); Education and autonomous rehabilitation instructions (33)	HI (VAS 0–100), HF (days/month)	Pressure pain threshold	20 applications over 4 weeks	Baseline, 4 weeks, 8 weeks
Haas et al. [55]	Cervical and thoracic manipulation—8 sessions (20); Cervical and thoracic manipulation—16 sessions (20)	High-velocity, low-amplitude spinal manipulation	Moist heat and light massage—8 sessions (20); Moist heat and light massage—16 sessions (20)	HI (MVK 0–100), HF (days/4 weeks)	Disability (MVK 0–10), Others headache frequency, Medications intake	8 or 16 sessions over 8 weeks. Groups undergoing 8 sessions also had 8 sessions of discussion and manual assessment	Baseline, 4 weeks, 8 weeks, 12 weeks, 16 weeks, 20 weeks, 24 weeks
Haas et al. [56]	Cervical and thoracic manipulation—6 sessions (65); Cervical and thoracic manipulation—12 sessions (64); Cervical and thoracic manipulation—18 sessions (63)	High-velocity, low-amplitude spinal manipulation	Sham massage (64)	HI (VAS 0–10), HF (days/4 weeks)	Disability (MVK), Drugs intake (N in the past 4 weeks), Other headache frequency (episodes in the past month)	6, 12 or 18 sessions over 6 weeks depending on group allocation. In the remaining visits, light massage was applied to experimental groups	Baseline, 6 weeks, 12 weeks, 18 weeks, 24 weeks, 39 weeks, 42 weeks
Hall et al. [57]	C1-2 SNAG (16)	The self-directed use of a self-SNAG strap to emphasize C1-2 rotation through passive motion	Sham SNAG (16)	Headache index (HI × HF × HD)	CROM (FRT), perceived benefit (VAS 0–10)	Daily self-administration for 12 months	Baseline, 4 weeks, 12 months
Jafari et al. [58]	Trigger point therapy (9)	Passive progressive manual pressure over trigger points	No treatment control (10)	HI (VAS 0–10), HF (days/2 weeks)	Change in elastic modulus in trigger point area (Pa), Trigger point area (cm2), Pressure pain threshold (Algometer, VAS), CGH duration (hrs in the last 2 weeks)	4 sessions over 8 days	Baseline, 3 weeks
Jull et al. [59]	Spinal mobilization and manipulation (51); low load endurance exercises for cervico-scapular muscle (52); Comination of the above (49)	Spinal manipulation: High-velocity, low-amplitude spinal manipulation Spinal mobilization: low-velocity spinal mobilization Low-load endurance exercise: a program of low-load exercises to train muscle control of the cervico-scapular region	No treatment control (48)	HI (VAS 0–10), HF (days/week)	Cervical pressure pain threshold (VAS), Deep Cervical flexors performance (CCFT), Pain with neck movement (VAS 0–10), Neck pain index (Northwick Park), CGH duration (hrs in the past week), Medication intake	8 to 12 sessions over 6 weeks	Baseline, 1 week, 3 months, 6 months, 12 months
Malo-Urriès et al. [60]	Upper cervical mobilization (41)	Low-velocity spinal mobilization	Control (lay supine for 30') (41)	HI (VAS 0–10)	General cervical spine ROM (using CROM device), Upper cervical spine ROM (using CROM device during FRT), Cervical, suboccipital, trapezius pressure pain threshold (Algometer, Kg/cm2)	Single intervention	Baseline, immediately after treatment
Nilsson et al. [61]	Cervical manipulation (28)	High-velocity, low-amplitude spinal manipulation	G2: Deep friction massage, trigger point therapy to upper back and neck, laser light therapy (26)	HI (VAS 0–100)	Medications intake	6 sessions over 3 weeks	Baseline; 5 weeks
Sedighi et al. [62]	Sub-occipital and trapezius dry needling (15)	An invasive intervention where a solid filament needle is inserted into a trigger point	Sham sub-occipital and trapezius dry needling (15)	Headache index (HI × HF)	Cervical spine ROM (4-points scale), Sub-occipital and trapezius pressure pain threshold (4-points scale, Function (FRI)	Single intervention	Baseline, post-intervention, 1 weeks
Sharma et al. [63]	Multimodal therapy (9); G2: exercise therapy (low load and mobility exercise program for the cervical spine, postural correction intervention) (9)	Multimodal therapy: spinal mobilization, low load and mobility exercise program for the cervical spine, postural correction intervention)	Postural correction (9)	HI (MVK 0–100), HF (days/week)	Deep Cervical flexors performance (CCFT), Disability (NDI), CGH duration (hrs in the past week)	12 sessions over 4 weeks	Baseline, 1 week, 2 weeks, 3 weeks, 4 weeks
von Piekartz et al. [64]	Manual therapy + exercise (22)	TMJ mobilization, trigger point therapy and stretching, TMJ coordination-ROM-home exercises	Usual care (cranio-cervical manual and exercise therapy) (21)	HI (CAS 0–10)	Anamnestic questionnaire (AQ), Graded-chronic pain status (GCPS-NL, scale 1 to 4), Temporo-mandibular pressure pain threshold (Digital algometer, Kgf), Disability (NDI), TMJ noise (presence/absence of click, TMJ-related disability (GCPS), TMJ ROM (cm), Pain with TMJ opening, Mandibular deviation (presence/absence)	6 sessions over 3–6 weeks	Baseline, 3 months, 6 months
Yang and Kang [65]	Hot pack and low frequency therapy on trapezius + cranio cervical flexion exercises (10); Hot pack and low frequency therapy on trapezius + sub-occipital relaxation (10)	Cranio-cervical exercise: low-intensity exercise aimed at recruiting deep cervical spine flexors painlessly Sub-occipital relaxation: light, prolonged manual pressure over the suboccipital muscles	Hot pack and low frequency therapy on trapezius (10)	HI (VAS 0–100)	Muscular fatigue (Hz), Muscle tone (N/m)	20 sessions over 4 weeks	Baseline, 2 weeks, 4 weeks
Youssef and Shanb [66]	Cervical mobilization and exercise (20)	Low-velocity spinal mobilization	Massage and exercise (18)	HI (VAS 0–10), HF (days/4 weeks)	Cervical spine ROM (cm), Disability (NDI), HD (hours/week)	12 sessions over 6 weeks	Baseline, 7 weeks
Abdel et al. [67]	Exercise Therapy (stretching, isometric contractions, postural correction) + Graston technique (30)	Graston technique: an instrument-assisted soft tissue mobilization	Exercise Therapy (stretching, isometric contractions, postural correction) (30)	HI (VAS 0–100), HF (days/week)	Disability (NDI), Cervical spine ROM (CROM device), HD (hours in the past week), Medication intake	12 sessions over 4 weeks	Baseline, 2 weeks, 4 weeks
Dunning et al. [68]	Spinal manipulation and electrical dry needling (74)	High-velocity, low-amplitude spinal manipulation Electrical dry needling: application of needles over the cervical, occipital, temporal and oculo-frontal areas with low-frequency electrostimulation	Spinal mobilization and scapular and cranio-cervical exercise (68)	HI (NPRS 0–10), HF (days/week)	HD (hours in the past week), Disability (NDI), Global Rating of Change (GROC), Medication intake	4 to 8 sessions over 4 weeks	Baseline, 1 week, 4 weeks, 3 months
Lerner-Lentz et al. [69]	Spinal manipulation, cranio-cervical and scapular exercise (24)	Spinal manipulation: High-velocity, low-amplitude spinal manipulation	Spinal mobilization and scapular and cranio-cervical exercise (21)	HI (NPRS 0–10)	Disability (NDI, HIT-6), GRC, Patient Acceptable Symptoms Scale (PASS)	Two sessions, uncertain timing	Baseline, 2 days, 1 week, 1 month
Moustafa et al. [70]	Myofascial release, spinal mobilization, cranio-cervical and scapular exercise, Dennerol cervical extension traction (30)	Dennerol cervical traction: the use of an orthotic device in supine position creating cervical spine traction	Myofascial release, spinal mobilization, cranio-cervical and scapular exercise (30)	HF (days/2 weeks)	Disability (HIT-6; HDI), Radiographic cervical alignment variables, Daily Defined Dose (DDD)	30 sessions over 10 weeks	Baseline, 10 weeks, 1 year, 2 years

*N* number of participants, *CCFT* cranio-cervical flexion test, *FRT* flexion-rotation test, *TMJ* temporo-mandibular joint, *HI* headache intensity, *HF* headache frequency, *ROM* range of motion, *HD* headache duration, *NDI* Neck Disability Index

As part of the inclusion criteria, all included trials described the diagnostic criteria used during their screening process. The official ICHD and CHISG diagnostic criteria [2, 3] were strictly followed by a limited number of studies, whilst the majority utilized modified versions of such criteria. In most cases, the discrepancy between the official sets of criteria and the ones used by the trials was the absence of diagnostic nerve blocks, which is a fundamental criterion for the CHISG, but not for the IHS.

### Risk of Bias

The RoB analysis showed high inter-rater reliability (Cohen’s Kappa = 0.87). Overall RoB was low in eight trials [53, 55, 56, 60, 61, 66–68], unsure in six trials [51, 58, 62–64, 69], and high in six trials [52, 54, 57, 59, 65, 70] for the primary and secondary outcome measures. Further detail regarding the RoB of individual studies is found in Table 2.

**Table 2:** Risk of Bias assessment of the included trials, according to the criteria by the Cochrane Back and Neck Group [19]

Study authors	1. Was the method of randomization adequate?	2. Was the treatment allocation concealed?	3. Was the patient blinded to the intervention?	4. Was the care provider blinded to the intervention?	5. Was the outcome assessor blinded to the intervention?	6. Was the drop out rate described and acceptable?	7. Were all randomized participants analyzed in the group to which they were allocated?	8. Are reports of the study free of suggestion of selective outcome reporting?	9. Were groups similar at regarding the most important prognostic indicators?	10. Were co-interventions avoided or similar?	11. Was the compliance acceptable in all groups?	12. Was the timing of the outcome assessment similar in all groups?	13. Are other sources of bias unlikely?	Overall risk of bias
Bodes-Pardo et al. [51]	Yes	Unsure	Unsure	No	Unsure	Yes	Yes	Yes	Yes	Yes	Yes	Yes	Yes	Unsure
Chaibi et al. [52]	Yes	Yes	No	No	No	No	Yes	Yes	No	Yes	Unsure	Yes	Yes	High
Dunning et al. [53]	Yes	Yes	No	No	No	Yes	Yes	Yes	Yes	Yes	Yes	Yes	Yes	Low
Esin et al. [54]	Yes	Unsure	Unsure	No	No	No	Unsure	Yes	Yes	Unsure	Unsure	Yes	Unsure	High
Haas et al. [55]	Yes	Yes	No	No	No	Yes	Yes	Yes	Yes	Yes	Yes	Yes	Yes	Low
Haas et al. [56]	Yes	Yes	No	No	No	Yes	Yes	Yes	Yes	Yes	Yes	Yes	Yes	Low
Hall et al. [57]	Yes	Unsure	Yes	No	Yes	Yes	Yes	Yes	Yes	No	Unsure	Yes	Yes	High
Jafari et al. [58]	Yes	Unsure	No	No	No	Yes	Yes	Yes	Yes	Yes	Unsure	Yes	Yes	Unsure
Jull et al. [59]	Yes	Unsure	No	No	No	Yes	Yes	Yes	Yes	No	Yes	Yes	Yes	High
Malo-Urriès et al. [60]	Yes	Yes	No	No	No	Yes	Yes	Yes	Yes	Yes	Yes	Yes	Yes	Low
Nilsson et al. [61]	Yes	Yes	No	No	No	Yes	Yes	Yes	Yes	Yes	Yes	Yes	Yes	Low
Sedighi et al. [62]	Unsure	Unsure	No	No	No	Yes	Yes	Yes	Yes	Yes	Yes	Yes	Yes	Unsure
Sharma et al. [63]	Yes	Unsure	No	No	No	Yes	Unsure	Yes	Unsure	Unsure	Unsure	Yes	Unsure	Unsure
von Piekartz et al. 2011 [64]	Yes	Yes	No	No	No	Yes	Yes	Yes	Yes	Unsure	Yes	Yes	Yes	Unsure
Yang and Kang [65]	Unsure	Unsure	No	No	No	No	Unsure	Yes	Unsure	Unsure	Unsure	Yes	Unsure	High
Youssef and Shanb [66]	Yes	Yes	No	No	No	Yes	Yes	Yes	Yes	Yes	Yes	Yes	Yes	Low
Abdel et al. [67]	Yes	Yes	No	No	No	Yes	Yes	Yes	Yes	Yes	Yes	Yes	Yes	Low
Dunning et al. [68]	Yes	Yes	No	No	No	Yes	Yes	Yes	Yes	Yes	Yes	Yes	Yes	Low
Lerner-Lentz et al. [69]	Yes	Yes	No	No	No	Yes	Yes	Yes	Yes	Yes	Yes	Unsure	Yes	Unsure
Moustafa et al. [70]	Yes	Yes	No	No	No	Yes	Yes	No	Yes	Yes	Yes	Yes	Yes	High

Studies were considered as overall low-RoB if no individual domain was rated as “high” or “unsure” RoB. Studies which scored “unsure”, but not “high”, for one or more items, were considered as overall unsure RoB, and “high” if any individual item was rated as high RoB

### Descriptive analysis: primary outcome measures

Among the included trials, the majority analyzed manual therapy in isolation: six focussed on spinal manipulation [52, 53, 55, 56, 61, 68], two on trigger point therapy [51, 58], two on spinal mobilization [57, 60], and one study each on kinesio-taping [54] and dry needling [62]. Seven trials used a combination of manual and exercise therapy [59, 63, 64, 66, 67, 69, 70], and two used exercise therapy alone [59, 65]. Ten studies used “other interventions” in their control groups (e.g. spinal mobilization, scapulo-thoracic exercises, trigger point therapy), nine studies used sham or placebo interventions, and four used no treatment. Nine studies had a long-term follow-up, and the last follow-ups among these studies averaged 42 weeks, ranging from 3 months to 2 years. Headache intensity was assessed with an 11 or 101-point Visual Analogue Scale (VAS), 11-point Numerical Pain Rating Scale (NPRS), 11-point Coloured Analogue Scale (CAS), and with a 100-point Modified Von Korff Scale. Composite headache questionnaires, which combined headache intensity, frequency, and other outcome measures, were used in two trials; these were not comparable to other pain intensity scales and relevant raw data could not be accessed [57, 62]. Headache frequency was assessed as the “number of days (with headache) in the previous four weeks”, “days in the previous two weeks”, or as “days in the previous week”. The following descriptive analysis is categorized according to the main study interventions and provides a brief overview of the findings from included trials. Tables 1 and 3 include further detail, list the statistical significance and MCIDs and should be referred to for a complete overview of the trials’ results.

**Table 3:** Primary outcome measures results

Study	Intervention (N)	Comparator (N)	HI scale	HI: MCID reached for Intervention group (Mean change at short term)	HI: MCID reached for Intervention group (Mean change at long term)	HF scale	HF: MCID reached for Intervention group (Mean change at short term)	HF: MCID reached for Intervention group (Mean change at long term)	*P* value between-groups	RoB
Bodes-Pardo et al. [51]	Trigger point therapy + stretching (10)	Sham trigger point therapy (10)	NPRS 0–10	MCID reached (− 5.4/10 at 1 week)					*P* < .001	Unsure
Chaibi et al. [52]	Cervical manipulation (4)	Sham manual therapy (4); Control (4)	VAS 0–10		MCID unknown (− 2.3 at 3 months)	Days/month		− 6 at 3 months	*P* values not calculated due to small sample size	High
Dunning et al. [53]	Cervical and thoracic manipulation (58)	Cervical and thoracic mobilization and cranio-cervical flexion exercises (52)	NPRS 0–10	MCID reached (− 4.5/10 at 4 weeks)	MCID reached (− 4.3/10 at 3 months)				*P* < .001 at all time points	Low
Esin et al. [54]	Kinesiotaping (34)	Sham taping (34); Education and autonomous rehabilitation instructions (33)	VAS 0–100	MCID unknown (− 2.3/100 at 4 weeks, − 29.5/100 at 8 weeks)		Days/month	MCID reached (− 8 at 4 weeks, − 9.7 at 8 weeks)		*P* < .01 for both comparisons at long term, and for HF at short term. *P* > 0.05 for HI at short term	High
Haas et al. [55]	Cervical and thoracic manipulation—8 sessions (20)	Moist heat and light massage—8 sessions (20)	MVK 0–100	MCID unknown (− 21/100 at 12 weeks)	MCID unknown (− 18/100 at 24 weeks)	Days/4 weeks	MCID reached (− 9 at 12 weeks)	MCID reched (− 7.6 at 24 weeks)	Analysis comparing both intervention groups ot both control groups found *P* < .05 for all outcomes at all time points, besides *P* > .05 for HF at 24 weeks	Low
	Cervical and thoracic manipulation—16 sessions (20)	Moist heat and light massage—16 sessions (20)	MVK 0–100	MCID unknown (− 21/100 at 12 weeks)	MCID unknown (− 23/100 at 24 weeks)	Days/4 weeks	MCID reached (− 9.6 at 12 weeks	MCID reched (− 9.4 at 24 weeks)		
Haas et al. [56]	Cervical and thoracic manipulation—6 sessions (65)	Sham massage (64)	VAS 0–10	MCID unknown (− .5/10 at 6 weeks)	MCID unknown (− .6/10 at 24 weeks)	Days/4 weeks	MCID not reached (− 3 at 6 weeks)	MCID not reached (− 5.2 at 24 weeks)	*P* > .05 at all time points	Low
	Cervical and thoracic manipulation—12 sessions (64)	Sham massage (64)	VAS 0–10	MCID unknown (− .7/10 at 6 weeks)	MCID unknown (− .7/10 at 24 weeks)	Days/4 weeks	MCID not reached (− 5 at 6 weeks)	MCID not reached (− 5.5 at 24 weeks)	*P* > .05 at all time points	
	Cervical and thoracic manipulation—18 sessions (63)	Sham massage (64)	VAS 0–10	MCID unknown (− .9/10 at 6 weeks)	MCID unknown (− .6/10 at 24 weeks)	Days/4 weeks	MCID not reached (− 7.7 at 6 weeks)	MCID not reached (− 7.2 at 24 weeks)	*P* < .05 at all time points for HF. *P* < .05 at 6 and 52 weeks for HI	
Hall et al. [57]	C1-2 SNAG (16)	Sham SNAG (16)	CGH index	MCID unknown (− 21/100 at 4 weeks)	MCID unknown (− 28/100 at 24 weeks)	CGH index	MCID unknown (− 21/100 at 4 weeks)	MCID unknown (− 28/100 at 12 months)	*P* < .001 at 4 weeks and 12 months	High
Jafari et al. [58]	Trigger point therapy (9)	No treatment control (10)	VAS 0–10	MCID unknown (− 2.4/10 at 3 weeks)		Days/2 weeks	MCID reached (− 1.8 at 3 weeks)		*P* < .05 at 3 weeks	Unsure
Jull et al. [59]	Spinal mobilization and manipulation (51)	No treatment control (48)	VAS 0–10	MCID unknown (− 3 at 7 weeks)	MCID unknown (− 2.3/10 at 12 months)	Days/week	MCID reached (− 2 at 7 weeks)	MCID reached (− 2.2 at 12 months)	*P* < .001 at 7 weeks for both outcomes, *P* < .05 at 12 months for HI and *P* < .01 for HF	High
	Low load endurance exercises for cervico-scapular muscle (52)	No treatment control (48)	VAS 0–10	MCID unknown (− 3.3 at 7 weeks)	MCID unknown (− 2.8/10 at 12 months)	Days/week	MCID reached (− 2.4 at 7 weeks)	MCID reched (− 2.5 at 12 months)	*P* < .001 for HF at all time points, *P* < .05 at 7 weeks and *P* < .01 at 12 months for HI	
	Combination of the above (49)	No treatment control (48)	VAS 0–10	MCID unknown (− 3.4 at 7 weeks)	MCID unknown (− 2.7/10 at 12 months)	Days/week	MCID reached (− 2 at 7 weeks)	MCID reched (− 2.1 at 12 months)	*P* < .001 at all time points	
Malo-Urriès et al. [60]	Upper cervical mobilization (41)	No treatment control (41)	VAS 0–10	MCID unknown (− .6 post TTT)					*P* < .05	Low
Nilsson et al. [61]	Cervical manipulation (28)	G2: Deep friction massage, trigger point therapy to upper back and neck, laser light therapy (26)	VAS 0–100	MCID unknown (− 16/100 at 5 weeks)					*P* < .05	Low
Sedighi et al. [62]	Sub-occipital and trapezius dry needling (15)	Sham sub-occipital and trapezius dry needling (15)	CGH index	MCID unknown (− 8.4 At 1 week)		CGH index	MCID unknown (− 8.4 At 1 week)		*P* > .05	Unsure
Sharma et al. [63]	Multimodal therapy (spinal mobilization, low load and mobility exercise program for the cervical spine, postural correction intervention) (9)	Postural correction (9)	VAS 0–100	MCID unknown (− 71 at 4 weeks)		Days/week	MCID reached (− 5.2 at 4 weeks)		*P* < .05 for both outcome measures	Unsure
	Exercise therapy (low load and mobility exercise program for the cervical spine, postural correction intervention) (9)	Postural correction (9)	VAS 0–100	MCID unknown (− 71 at 4 weeks)		Days/week	MCID reached (− 5.2 at 4 weeks)		*P* < .05 for HI, *P* > .05 for HF	
von Piekartz et al. [64]	Manual therapy + exercise (TMJ manual therapy, TMJ coordination and ROM exercises) (22)	Usual care (cranio-cervical manual and exercise therapy) (21)	CAS 0–10		MCID unknown (− 3,9 at 3 months, − 4.9 at 6 months)				*P* < .001 at all time points	Unsure
Yang and Kang [65]	Hot pack and low frequency therapy on trapezius + cranio cervical flexion exercises (10)	Hot pack and low frequency therapy on trapezius (10)	VAS 0–100	MCID unknown (unclear results at 4 weeks)					*P* < .05 reported despite wrong results reported at weel 4	High
	Hot pack and low frequency therapy on trapezius + sub-occipital relaxation (10)	Hot pack and low frequency therapy on trapezius (10)	VAS 0–100	MCID unknown (wrong 4 weeks measure					*P* < .05 reported despite wrong results reported at weel 4	
Youssef and Shanb [66]	Cervical mobilization and exercise (20)	Massage and exercise (18)	VAS 0–10	MCID unknown (− 4.9 at 7 weeks)		Days/4 weeks	MCID reached (− 4.1 at 7 weeks)		*P* < .05 for both outcome measures	Low
Abdel et al. [67]	Exercise Therapy (stretching, isometric contractions, postural correction) + Graston technique (30)	Exercise Therapy (stretching, isometric contractions, postural correction) (30)	VAS 0–100	MCID unknown (MD = 36.7/100 at 4 weeks)		Days/week	Values expressed in median, difference of median for the Intervention group reaching MCID (− 80% from baseline)		*P* = 0.0001 for HI, *P* = 0.001 for HF	Low
Dunning et al. [68]	Spinal manipulation and electrical dry needling (74)	Spinal mobilization and scapular and cranio-cervical exercise (68)	NPRS 0–10	MCID reached (MD = 3.9 at 4 weeks)	MCID reached (MD = 4.9 at 3 months)	Days/week	MCID reached (MD = 2.9 at 4 weeks)	MCID reached (MD = 3.5 at 3 months)	*P* < 0.001 at 4 weeks and 3 months for HI and HF	Low
Lerner-Lentz et al. [69]	Spinal manipulation, cranio-cervical and scapular exercise (21)	Spinal mobilization and scapular and cranio-cervical exercise (24)	NPRS 0–10	MCID reached (MD = 4.3 at 1 month)					*P* > 0.05 at all time points. Within-group *P* < 0.05 for both groups at all timepoints	Unsure
Moustafa et al. [70]	Myofascial release, spinal mobilization, cranio-cervical and scapular exercise, Dennerol cervical extension traction (30)	Myofascial release, spinal mobilization, cranio-cervical and scapular exercise (30)				Days/2 weeks	MCID reached (MD = 5.5 at 10 weeks)	MCID reached (MD = 10.4 at 1 year)	*P* < 0.001 at all time points for HF	High

For each trial, the intervention and comparator(s), the number of participants for each group, the measurement scales used for HI and HF, the RoB score, and mean changes with statistical and clinical significance values (*P* value and MCID) at each time-point are described

*N* number of participants, *TMJ* temporo-mandibular joint, *HI* headache intensity, *HF* headache frequency, *MCID* Minimal Clinically Important Difference

Only 8 of the included trials reported whether adverse events were monitored. No severe adverse events were reported, but minor or transient adverse effects were noted in 3 trials [59, 64, 68], which are described in Table 4.

**Table 4:** Adverse events reported by each trial are described

Study	Adverse events
Bodes-Pardo et al. [51]	Not reported
Chaibi et al. [52]	No severe or serious adverse effects
Dunning et al. [53]	No severe or serious adverse effects
Esin et al. [54]	Not reported
Haas et al. [55]	Not reported
Haas et al. [56]	No severe or serious adverse effects
Hall et al. [57]	Not reported
Jafari et al. [58]	Not reported
Jull et al. [59]	No severe or serious adverse effects. 6.7% of total headaches experienced by participants during the trial were reported to be caused by the treatment
Malo-Urriès et al. [60]	No severe or serious adverse effects
Nilsson et al. [61]	Not reported
Sedighi et al. [62]	Not reported
Sharma et al. [63]	Not reported
von Piekartz et al. [64]	No severe or serious adverse effects. 3 patients dropped out after the second follow up due to worsening of their symptoms
Yang and Kang [65]	Not reported
Youssef and Shanb [66]	Not reported
Abdel et al. [67]	Not reported
Dunning et al. [68]	No severe or serious adverse effects. 60% of participants in the dry needling group experienced localized soreness, and 24% localized ecchymosis, resolved withing 48 h. 4% experienced drowsiness or nausea, resolved within several hours
Lerner-Lentz et al. [69]	Not reported
Moustafa et al. [70]	No severe or serious adverse effects

#### Spinal manipulation

Overall, 8 trials assessed the effectiveness of spinal manipulation. Two trials with low RoB [56, 57] (n = 336) compared spinal manipulation alone to sham treatments and found statistically significant changes in favor of spinal manipulation (*p* < 0.05) at short and long term. MCIDs for headache intensity and frequency were reached by one trial only [56], but over half of the participants receiving a higher dose of spinal manipulation achieved at least a 50% improvement in such outcomes in the second trial [57].

Three trials with low RoB (n = 306) compared spinal manipulation to other forms of manual therapy [53, 61, 68]. Spinal manipulation was found more effective than spinal mobilization and cranio-cervical flexion exercises (*p* < 0.001) [53], and multimodal therapy (deep friction massage, trigger point therapy, light laser therapy) (*p* < 0.05) [61], and MCIDs were reached at short and long term. A combination of spinal manipulation and electrical dry needling was found more effective than spinal mobilization and cranio-cervical exercises at short and long-term [68]. Important clinical changes were also found in favor of spinal manipulation (with or without exercise therapy] for headache frequency and intensity in two high and unsure-RoB trials (n = 245) [59, 69] at short and long term.

#### Spinal mobilization

The effectiveness of spinal mobilization was assessed by two trials with low RoB [60, 66] (n = 120) and one study with unsure RoB [63] (n = 36). Spinal mobilization (with or without exercise therapy) was found more effective than no-treatment [60], massage and exercise therapy [66], and postural correction or exercise therapy [63] (*p* < 0.05) at short term. For outcome measures with MCIDs available from the literature, MCIDs were reached within four to seven weeks in all trials.

#### Myofascial trigger point therapy

Two trials with small sample sizes (n = 38), unsure RoB and no long-term follow-up found statistically significant superiority of sternocleidomastoid myofascial trigger point release for CGH compared to sham trigger point therapy (*p* < 0.001), and a no treatment control (*p* < 0.05) [51, 58]. MCIDs for headache intensity and frequency were reached.

#### Dry needling

The unsure-RoB trial by Sedighi et al. [62] (n = 30) found no statistically significant changes (*p* > 0.05) for sub-occipital and trapezius dry needling compared to sham acupuncture at 1 week.

#### Temporo-mandibular joint (TMJ) treatment

One trial with unsure RoB (n = 43) [64] compared a similar set of manual and exercise therapy interventions (mobilization, trigger point release, coordination and stretching exercises depending on the therapists’ clinical decision) either directed to the TMJ area or to the cranio-cervical region in people living with CGH and showing signs of TMJ dysfunction. They found superior effects for the TMJ group (*p* < 0.001) at 6 months.

#### Kinesio-taping

Kinesio-taping was compared to sham taping and to home rehabilitation by one high-RoB trial [54] (n = 101), and statistical (*p* < 0.01) and clinical improvements at 4 and 8 weeks were reported. The study population consisted of teenagers aged 14–16 diagnosed with CGH and with presence of cervical “myogenic trigger zones”.

#### Therapeutic exercise

Two high-RoB trials (N = 140) assessed the effectiveness of therapeutic exercise in isolation. Jull et al. [59] compared low-load endurance cervico-scapular exercises to no treatment, and found statistical significant changes in headache intensity and frequency at 7 weeks and 12 months (*p* < 0.05). MCIDs were reached for headache frequency.

The high-RoB trial by Yang and Kang [65] (n = 30) compared cranio-cervical flexion exercises alone and manual suboccipital manual relaxation alone to a no-treatment control group. Despite between-group differences in headache intensity reported as significant (*p* < 0.05), the values reported in the study for the follow-up assessment were unequivocally mistaken (values of > 350 for a 0–100 VAS). The authors of the trial were contacted without success.

#### Self-sustained Natural Apophyseal Glide (SNAG)

Hall et al. [57] (n = 32) compared SNAG treatment to sham-SNAG. Patients were asked to perform SNAG autonomously twice daily for twelve months. A headache index was used as primary outcome measure, and significant between-group differences were found in favour of the experimental group at 4 weeks and twelve months (*p* < 0.05). Poor treatment compliance in the control group at four weeks, and in both groups at twelve months was reported, and the study had high RoB.

#### Graston technique

The low-RoB trial by Abdel et al. [67] (n = 60) compared Graston mobilization plus therapeutic exercise to exercise alone, and found between-group differences favoring Graston mobilization for headache intensity and frequency (*p* < 0.001) at four weeks. MCIDs for headache frequency were reached at 4 weeks.

#### Dennerol cervical extension traction

The high-RoB trial by Moustafa et al. [70] (n = 60) compared two groups treated with a mix of manual and exercise therapy, where the experimental group was also treated using the Dennerol traction device. The experimental group had significant improvements (*p* < 0.001) compared to the control group at ten weeks, one and two years for headache frequency, which also reached the MCID at all timepoints.

### Descriptive analysis: secondary outcome measures

Table 5 shows the results for the other outcome measures considered by each of the included studies, reporting levels of statistical and clinical significance when available, and the reader is invited to consult it for a more precise interpretation of the following section. The most common additional outcome measures used by the RCTs and included in this systematic review were disability (eleven trials), headache duration (eight trials) and pressure-pain-thresholds (seven trials). Cervical spine range of motion (CROM) and Medication intake were assessed in six trials, perceived change in four trials, and cervical flexors performance in three trials. A descriptive description of secondary outcome measures of interest (headache duration and disability) is provided in Table 5.

**Table 5:** Secondary outcome measures results

Study	Intervention (N)	Comparator (N)	Secondary outcome measures	Key findings
Bodes-Pardo et al. [51]	Trigger point therapy + stretching (10)	Sham trigger point therapy (10)	Neck pain intensity (VAS), Cervical spine ROM (CROM device), Cervical pressure pain threshold (algometer), Deep Cervical flexors performance (CCFT)	*P* < 0.001 for neck pain intensity, CCFT, pressure pain thresholds and CROM at 1 week. MCID reached for CROM (MD = 8.2–13.6°)
Chaibi et al. [52]	Cervical manipulation (4)	Sham manual therapy (4); Control (4)	CGH duration (hrs/day)	*P *values not calculated due to small sample size
Dunning et al. [53]	Cervical and thoracic manipulation (58)	Cervical and thoracic mobilization and cranio-cervical flexion exercises (52)	CGH duration (hrs in the last week), Disability (NDI), Global Rating of Change scale (GRC), Drugs intake	*P* < 0.001 for NDI and GRC at 1, 4 weeks and 3 months. *P* < 0.05 for headache duration at 1 week and 3 months. *P* < 0.001 for medication intake at 3 months. MCID proposed by the study for NDI were reached (MD = − 11,6 at 4 weeks)
Esin et al. [54]	Kinesiotaping (34)	Sham taping (34); Education and autonomous rehabilitation instructions (33)	Pressure pain threshold (cervical, sub-occipital, trapezius muscles)	*P* < 0.01 for pressure pain threshold at 4 and 8 weeks
Haas et al. [55]	Cervical and thoracic manipulation—8 sessions (20)	Moist heat and light massage—8 sessions (20)	Disability (MVK), Drugs intake (N in the past 4 weeks), Other headache frequency (episodes in the past month)	Intervention main effect: *P* < 0.05 for other headache frequency and medication intake at 24 weeks. *P* < 0.05 for Disability at 12 weeks
	Cervical and thoracic manipulation—16 sessions (20)	Moist heat and light massage—16 sessions (20)	Disability (MVK), Drugs intake (N in the past 4 weeks), Other headache frequency (episodes in the past month)	Intervention main effect: *P* < 0.05 for other headache frequency and medication intake at 24 weeks. *P* < 0.05 for Disability at 12 weeks
Haas et al. [56]	Cervical and thoracic manipulation—6 sessions (65)	Sham massage (64)	Disability (HIT-6), Perceived change (0-points scale), Quality of life (VAS 0–100), Subjective CGH improvement (20-points scale), satisfaction (7-points Likert scale), neck pain frequency	*P* < 0.05 for Disability at 6 and 24 weeks
	Cervical and thoracic manipulation—12 sessions (64)	Sham massage (64)	Disability (HIT-6), Perceived change (0-points scale), Quality of life (VAS 0–100), Subjective CGH improvement (20-points scale), satisfaction (7-points Likert scale), neck pain frequency	*P* < 0.05 for Disability, neck pain frequency and intensity at 6 weeks. *P* < 0.05 for perceived change at 6 and 24 weeks
	Cervical and thoracic manipulation—18 sessions (63)	Sham massage (64)	Disability (HIT-6), Perceived change (0-points scale), Quality of life (VAS 0–100), Subjective CGH improvement (20-points scale), satisfaction (7-points Likert scale), neck pain frequency	*P* < 0.05 for Disability, perceived change and neck pain frequency at 6 and 24 weeks. *P* < 0.05 for neck pain intensity at 6 weeks
Hall et al. [57]	C1-2 SNAG (16)	Sham SNAG (16)	Cervical spine ROM (CROM device during FRT)	*P* < 0.001 for CROM (FRT), MCID reached (MD = 11–20°) after 1st intervention
Jafari et al. [58]	Trigger point therapy (9)	No treatment control (10)	Change in elastic modulus in trigger point area (Pa), Trigger point area (cm2), Pressure pain threshold (Algometer, VAS), CGH duration (hrs in the last 2 weeks)	*P* < 0.05 for headache duration, pressure pain threshold, TrP area at 3 weeks
Jull et al. [59]	Spinal mobilization and manipulation (51)	No treatment control (48)	Cervical pressure pain threshold (VAS), Deep Cervical flexors performance (CCFT), Pain with neck movement (VAS), Neck pain index (Northwick Park), CGH duration (hrs in the past week), Medication intake	*P* < 0.05 for neck pain index, headache duration and pain on palpation at 7 weeks and 12 months. *P* < 0.05 for pain with neck movements at 7 weeks. *P* < 0.015 for medication intake at 12 months. *P* > 0.05 for CCFT at 7 weeks and 12 months
	Low load endurance exercises for cervico-scapular muscle (52)	No treatment control (48)	Cervical pressure pain threshold (VAS), Deep Cervical flexors performance (CCFT), Pain with neck movement (VAS), Neck pain index (Northwick Park), CGH duration (hrs in the past week)	*P* < 0.05 for neck pain index and pain with neck movement at 7 weeks and 12 months. *P* < 0.05 for pain on palpation at 7 weeks. *P* < 0.015 for medication intake at 12 months. *P* < 0.001 for CCFT at 7 weeks and 12 months
	Combination of the above (49)	No treatment control (48)	Cervical pressure pain threshold (VAS), Deep Cervical flexors performance (CCFT), Pain with neck movement (VAS), Neck pain index (Northwick Park), CGH duration (hrs in the past week)	*P* < 0.05 for neck pain index, headache duration and pain on palpation at 7 weeks and 12 months. *P* < 0.05 for pain with neck movements at 7 weeks. *P* < 0.015 for medication intake at 12 months. *P* < 0.001 for CCFT at 7 weeks, *P* < 0.01 at 12 months
Malo-Urriès et al. [60]	Upper cervical mobilization (41)	No treatment control (41)	General cervical spine ROM (using CROM device), Upper cervical spine ROM (using CROM device during FRT), Cervical, suboccipital, trapezius pressure pain threshold (Algometer, Kg/cm2)	*P* > 0.05 for pressure pain thresholds. *P* < 0.05 for general cervical ROM (flexion, left rotation), *P* = 0.006 for right FRT and P < 0.001 for left FRT post intervention. MCIDs for FRT were reached (MD + 5.4° to 7.4°)
Nilsson et al. [61]	Cervical manipulation (28)	G2: Deep friction massage, trigger point therapy to upper back and neck, laser light therapy (26)	Medication intake (N/day)	*P* = 0.14 at week 5, with P < 0.05 after X2 test for type-2 error. Within-group changes saw P < 0.0001 in the experimental group
Sedighi et al. [62]	Sub-occipital and trapezius dry needling (15)	Sham sub-occipital and trapezius dry needling (15)	Cervical spine ROM (4-points scale), Sub-occipital and trapezius pressure pain threshold (4-points scale), Function (FRI)	*P* < 0.01 for FRI at 1 week. Within-group changes only were provided for other outcomes, with P < 0.05 for ROM and P < 0.001 for trigger point tenderness in the MT group at 1 week
Sharma et al. [63]	Multimodal therapy (spinal mobilization, low load and mobility exercise program for the cervical spine, postural correction intervention) (9)	Postural correction (9)	Deep Cervical flexors performance (CCFT), Disability (NDI), CGH duration (hrs in the past week)	*P* = 0.001 for all outcome measures at 4 weeks. NDI MCID cannot be assessed due to absence of raw data
	Exercise therapy (low load and mobility exercise program for the cervical spine, postural correction intervention) (9)	Postural correction (9)	Deep Cervical flexors performance (CCFT), Disability (NDI), CGH duration (hrs in the past week)	*P* = 0.001 for all outcome measures at 4 weeks. NDI MCID cannot be assessed due to absence of raw data
von Piekartz et al. [64]	Manual therapy + exercise (TMJ manual therapy, TMJ coordination and ROM exercises) (22)	Usual care (cranio-cervical manual and exercise therapy) (21)	Anamnestic questionnaire (AQ), Graded-chronic pain status (GCPS-NL, scale 1 to 4), Temporo-mandibular pressure pain threshold (Digital algometer, Kgf), Disability (NDI), TMJ noise (presence/absence of click), TMJ-related disability (GCPS), TMJ ROM (cm), Pain with TMJ opening, Mandibular deviation (presence/absence)	Between-group *P* < 0.05 for all outcomes at 3 months. *P* < 0.05 for AQ, GCPS, pressure pain thresholds, TMJ noise, pain with TMJ opening at 6 months. MCID proposed by the study reached for NDI (− 6.5 at 3 months), not for mouth opening ROM at both timepoints
Yang and Shanb [65]	Hot pack and low frequency therapy on trapezius + cranio cervical flexion exercises (10)	Hot pack and low frequency therapy on trapezius (10)	Muscular fatigue (Hz), Muscle tone (N/m)	*P* < 0.05 for change in muscle fatigue for SCM and UFT muscles at 2 and 4 weeks. *P* < 0.05 for change in muscle tone at left UT and bilateral SCM at 2 and 4 weeks
	Hot pack and low frequency therapy on trapezius + sub-occipital relaxation (10)	Hot pack and low frequency therapy on trapezius (10)	Muscular fatigue (Hz), Muscle tone (N/m)	*P* < 0.05 for change in muscle fatigue for SCM and UFT muscles at 2 and 4 weeks. *P* < 0.05 for change in muscle tone at left UT and bilateral SCM at 2 and 4 weeks
Youssef and Shanb [66]	Cervical mobilization and exercise (20)	Massage and exercise (18)	Cervical spine ROM (cm), Disability (NDI), CGH duration (hours/week)	*P* < 0.05 for all ROM directions and CGH duration at 7 weeks. Between-group *P* = 0.26 for NDI at 7 weeks, but within-group *P* < 0.001 and MCID were reached (− 27.2 at 7 weeks)
Abdel et al. [67]	Exercise Therapy (stretching, isometric contractions, postural correction) + Graston technique (30)	Exercise Therapy (stretching, isometric contractions, postural correction) (30)	Disability (NDI), Cervical spine ROM (CROM device), HD (hours in the past week), Medication intake	Within-group *P* < 0.05 for flexion, left lateral flexion and right lateral flexion CROM at 2 weeks in the conrol group; *P* < 0.0001 for all other outcomes at all timepoints in both groups, and for between-groups differences at 4 weeks favouring Graston technique. MCIDs reached for NDI (MD = 11.1 at 4 weeks)
Dunning et al. [68]	Spinal manipulation and electrical dry needling (74)	Spinal mobilization and scapular and cranio-cervical exercise (68)	HD (hours in the past week), Disability (NDI), Global Rating of Change (GROC), Medication intake	Between-group *P* < 0.001 for HD at all timepoints, for GRC and Medication intake at 3 months. MCIDs reached for NDI (MD = 14.4 at 4 weeks and 16.9 at 3 months) and between-group *P* < 0.001 at all timepoints
Lerner-Lentz et al. [69]	Spinal manipulation, cranio-cervical and scapular exercise (21)	Spinal mobilization and scapular and cranio-cervical exercise (24)	Disability (NDI), Headache Impact Test (HIT-6), Globale Rate of Change (GRC), Patient Acceptable Symptoms Scale (PASS)	MCIDs reached for NDI in both groups (MD = 13.9 for manipulation and MD = 12.8 for mobilization group at 1 month). Within-group *P* < 0.05 at all timepoionts for all groups for all secondary outcomes. Between-groups *P* > 0.05 at all time points
Moustafa et al. [70]	Myofascial release, spinal mobilization, cranio-cervical and scapular exercise, Dennerol cervical extension traction (30)	Myofascial release, spinal mobilization, cranio-cervical and scapular exercise (30)	Disability (HIT-6; HDI), Radiographic cervical alignment variables, Daily Defined Dose (DDD)	Within-group *P* < 0.001 for HIT-6, HDI, DDD at 10 weeks for the control group, and at all time points for the intervention group. Between group *P* > 0.05 at 10 weeks for HIT-6, HDI, DDD. Between group *P* < 0.001 at all timepoints favouring the experimental group for Radiographic variables, and for all other outcome measures at 1 and 2-years follow ups

For each trial, the intervention and comparator(s), the number of participants for each group, the secondary outcome measures of interest, the RoB score, and mean changes with statistical and clinical significance values (P value and MCID) at each time-point are described.

*N* number of participants, *CCFT* cranio-cervical flexion test, *FRT* flexion-rotation test, *TMJ* temporo-mandibular joint, *ROM* range of motion, *HD* headache duration, *NDI* neck disability index, *MCID* minimal clinically important difference

#### Disability

When disability was measured with the NDI, five studies [53, 63, 64, 67, 68] found significant within- and between-group differences favoring experimental interventions (*p* < 0.05) when compared to “other interventions”. MCIDs were reached in four trials [53, 64, 67, 68], whilst the absence of raw data did not permit analysis of the fifth trial [63].

Youssef and Shanb and Lerner-Lentz et al. [66, 69] did not find significant between-group differences, although all groups involved in this study had a significant within-group improvement (*p* < 0.001 and *p* < 0.05 respectively) and reached the MCID for the NDI.

The two trials by Haas et al. [55, 56] found significant differences favouring spinal manipulation over sham manipulation at 6, 12 and 24 weeks (*p* < 0.05).

Sedighi et al. [62] found a greater efficacy of dry needling over sham acupuncture at one week after a single application (*p* < 0.001).

#### Headache Duration

Headache duration was measured as hours with headache per day or per week. The 2016 and 2018 trials by Dunning et al. [53, 68] found significant improvements after spinal manipulation at one week, four weeks and three months (*p* < 0.05). Jafari et al. [58] found effectiveness of trigger point therapy in decreasing headache duration at three weeks (*p* < 0.05). Jull et al. [59] found manual therapy with or without exercise therapy to be more effective than no treatment for headache duration at seven weeks and twelve months (*p* < 0.05), but low-load endurance exercise was not statistically more beneficial than no treatment (*p* > 0.05). Sharma et al. [63] found significant effects of mobilization and low-level exercise compared to postural correction and endurance exercise (*p* = 0.001) at four weeks. Significant improvements (*p* < 0.05) were also found for the experimental group by Youssef and Shanb [66], comparing cervical mobilization to massage therapy. Graston mobilization were found more effective than therapeutic exercise at four weeks for headache duration (*p* < 0.001) by Abdel et al. [67].

### Meta-analysis

Due to the various differences in the design of included trials, only six studies were deemed comparable in a meta-analysis [51, 52, 54–56, 62]. Specifically, data pooling was only possible for trials with sham controls, as not enough studies were comparing interventions to no-treatment controls or to other active interventions. For the pooled trials, meta-analysis was feasible for headache intensity and frequency both at short and long-term, and for disability at short-term.

**Fig. 4:**
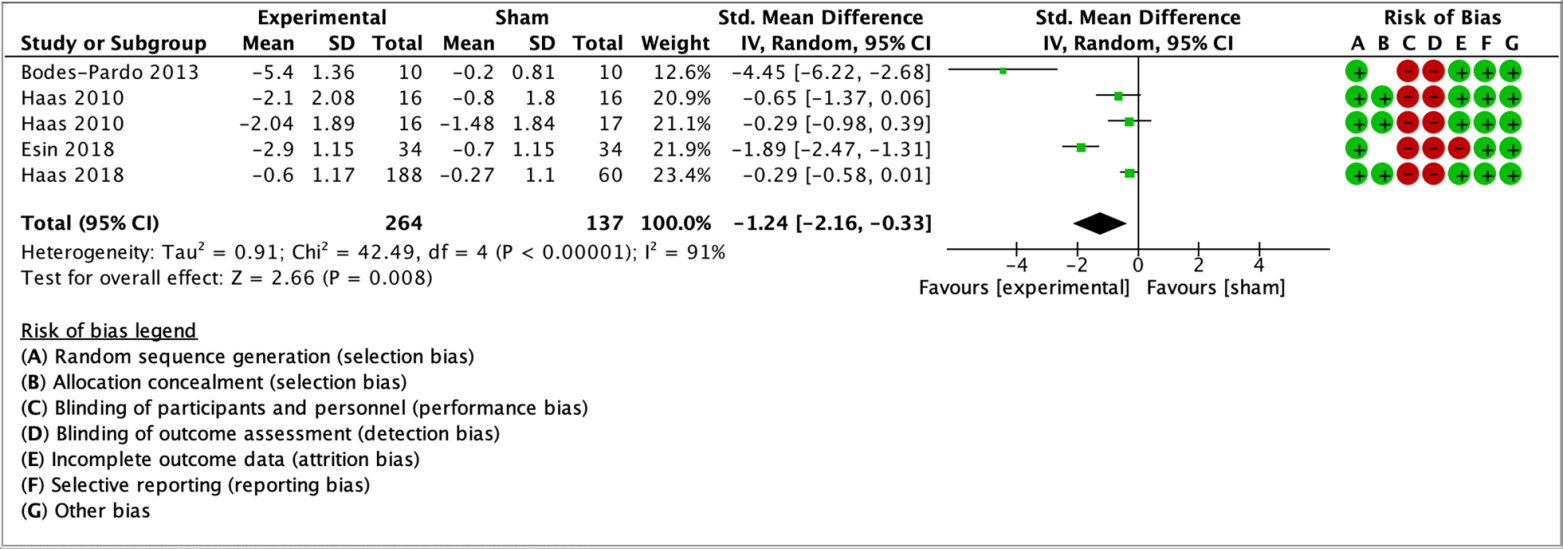
Forest plot for sham-controlled manual therapy trials assessing headache intensity at short term (< 3 months). Outcome measures are reported as standardized mean changes with RoB assessment for each study

**Fig. 5:**
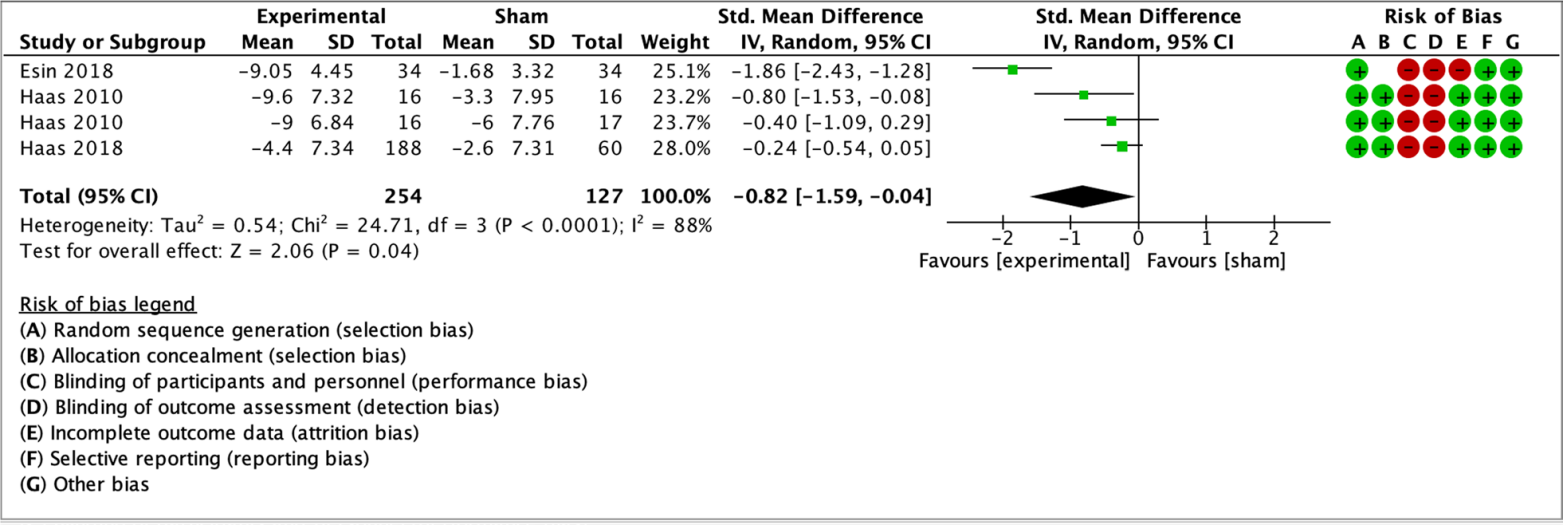
Forest plot for sham-controlled manual therapy trials assessing headache frequency at short term (< 3 months). Outcome measures are reported as standardized mean changes with RoB assessment for each study

**Fig. 6:**
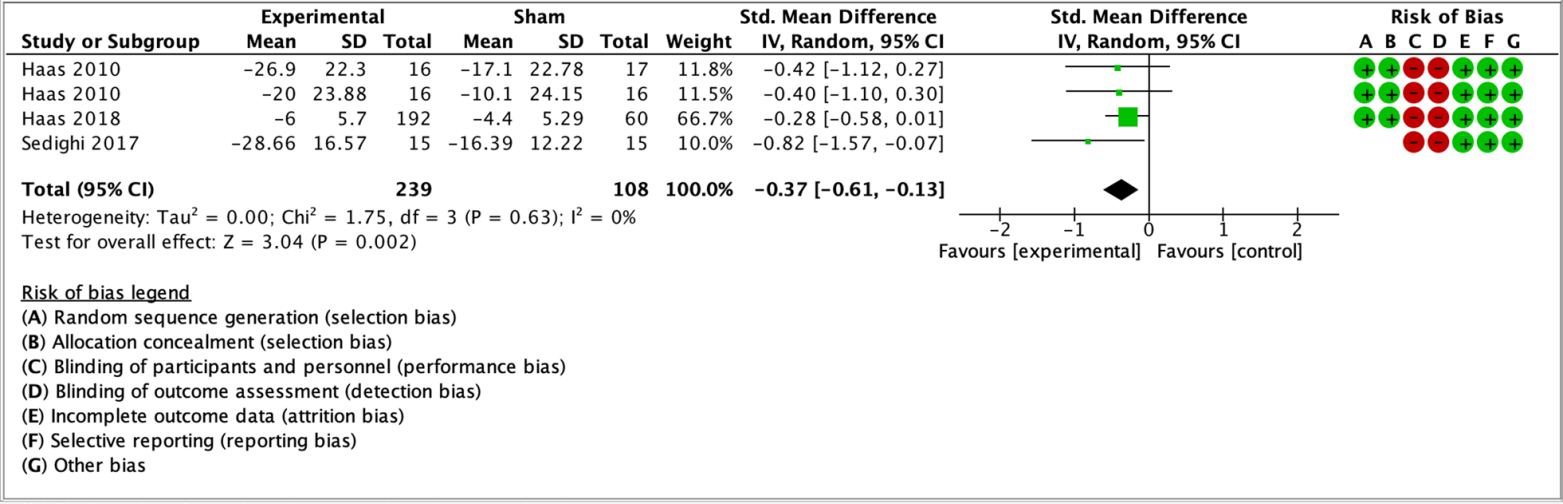
Forest plot for sham-controlled manual therapy trials assessing disability at short term (< 3 months). Outcome measures are reported as standardized mean changes with RoB assessment for each study

**Fig. 7:**
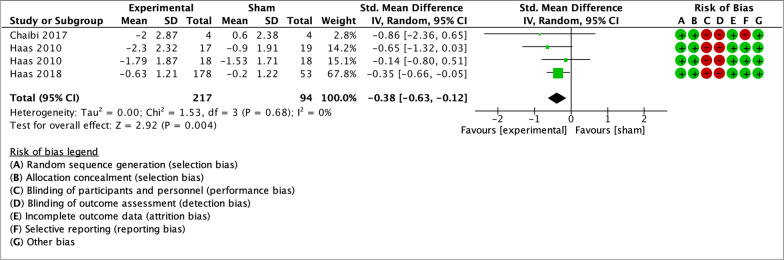
Forest plot for sham-controlled manual therapy trials assessing headache intensity at long term (> 3 months). Outcome measures are reported as standardized mean changes with RoB assessment for each study

**Fig. 8:**
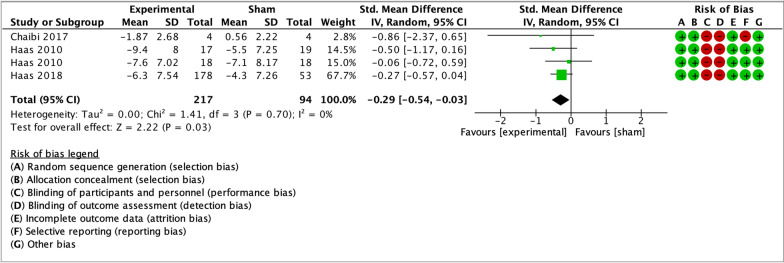
Forest plot for sham-controlled manual therapy trials assessing headache frequency at long term (> 3 months). Outcome measures are reported as standardized mean changes with RoB assessment for each study

**Fig. 9:**
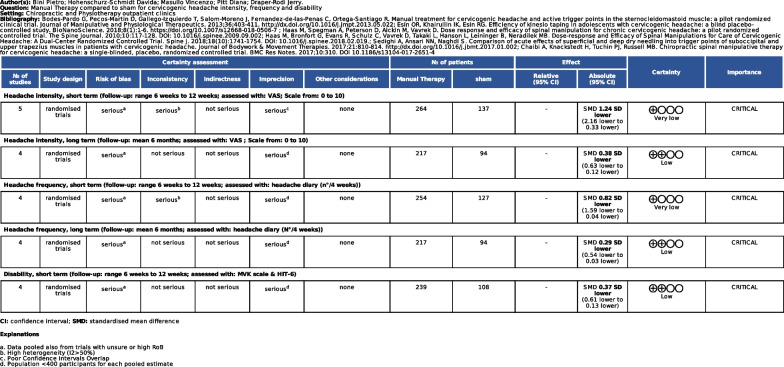
The summary of findings describes the overall quality of evidence for each outcome measure considered following GRADE assessment, with justifications for downgrading each pooled estimate [33]. Studies included in this analysis were pooled regardless of their RoB assessment

As illustrated by the forest plots (Figs. 4, 5, 6, 7 and 8), a large effect was found in favour of manual therapy for headache intensity and moderate-to-large effects for headache frequency at short-term. For disability, there was a small-to-moderate effect at short-term. Long-term effects were small-to-moderate for headache intensity and small for headache frequency. The GRADE assessment for the quality of evidence showed very low quality of evidence for Headache Intensity and Frequency at short term (downgraded due to risk of bias, inconsistency, and imprecision), and low quality of evidence for Headache intensity, frequency and disability at long term (downgraded due to risk of bias and imprecision). As none of the comparisons included 10 or more studies, publication bias could not be assessed [38]. The summary of findings table can be found in Fig. 9.

**Fig. 10:**
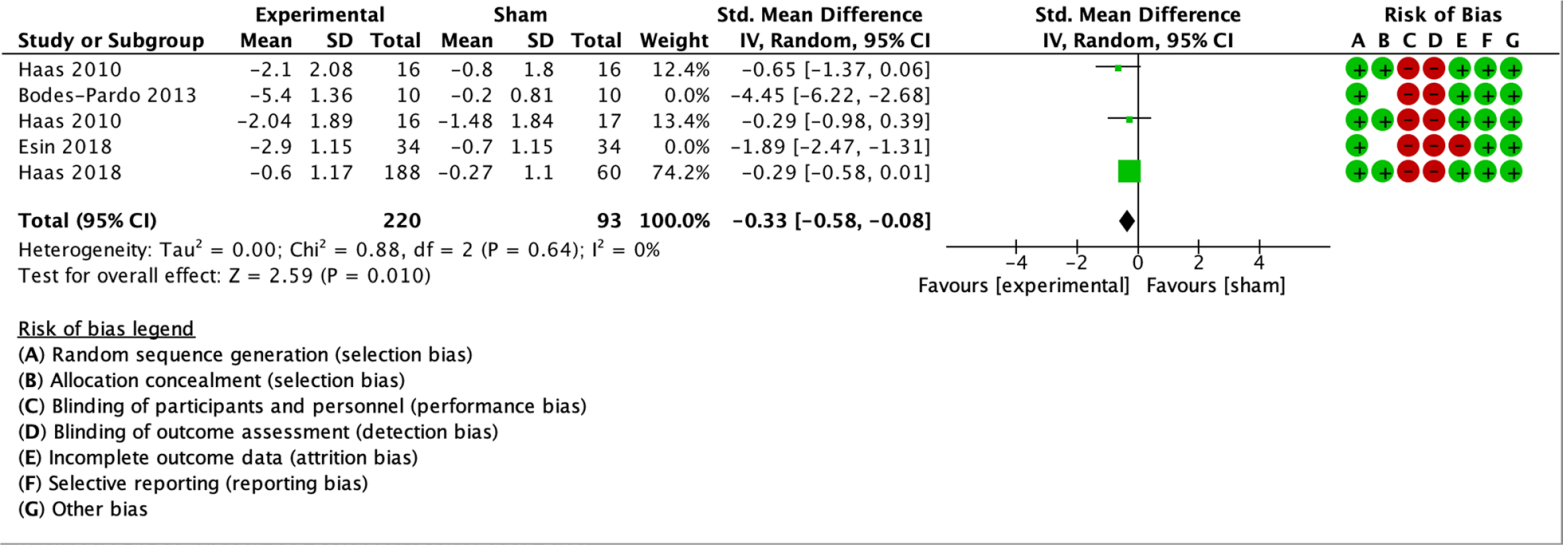
Forest plot for sham-controlled, low-RoB manual therapy trials assessing headache intensity at short term (< 3 months). Outcome measures are reported as standardized mean changes with RoB assessment for each study

**Fig. 11:**
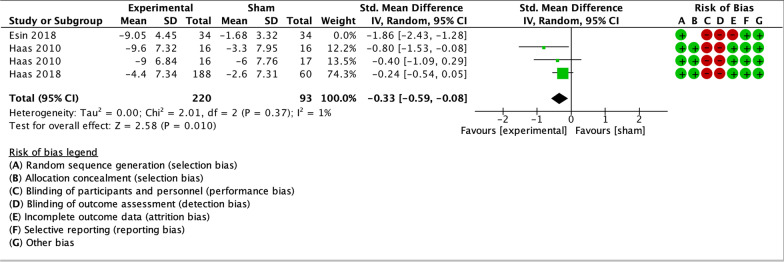
Forest plot for sham-controlled, low-RoB manual therapy trials assessing headache frequency at short term (< 3 months). Outcome measures are reported as standardized mean changes with RoB assessment for each study

**Fig. 12:**
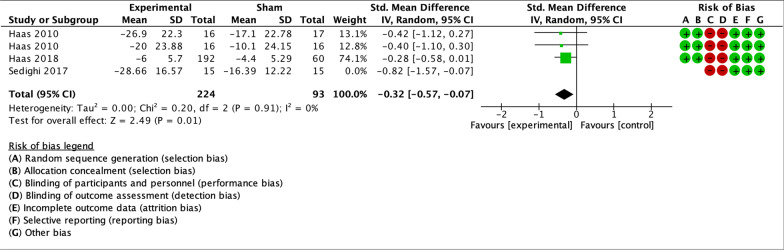
Forest plot for sham-controlled, low-RoB manual therapy trials assessing disability at short term (< 3 months). Outcome measures are reported as standardized mean changes with RoB assessment for each study

**Fig. 13:**
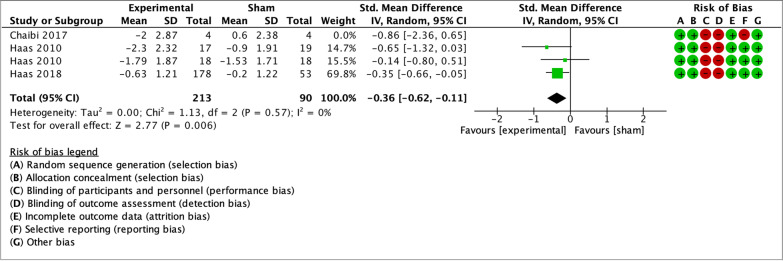
Forest plot for sham-controlled, low-RoB manual therapy trials assessing headache intensity at long term (> 3 months). Outcome measures are reported as standardized mean changes with RoB assessment for each study

**Fig. 14:**
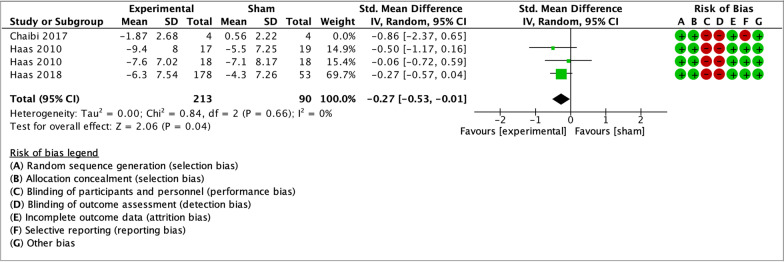
Forest plot for sham-controlled, low-RoB manual therapy trials assessing headache frequency at long term (> 3 months). Outcome measures are reported as standardized mean changes with RoB assessment for each study

**Fig. 15:**
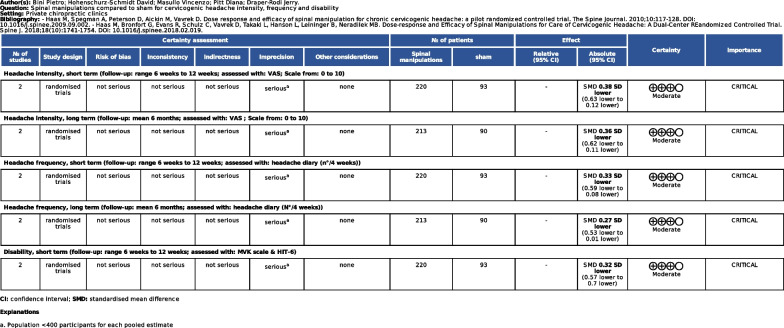
The summary of findings describes the overall quality of evidence for each outcome measure following GRADE assessment, with justifications for downgrading each pooled estimate [33]. Only low-RoB studies were pooled for this analysis

Only two trials in the meta-analysis had a low RoB for primary and secondary outcome measures, and both analyzed spinal manipulation. A sensitivity analysis including only these two studies was performed. The trials included groups with different dosages of the same intervention as parallel experimental groups. Haas et al. [55] contributed to data pooling with two comparisons: manipulation vs sham (8 sessions) and manipulation vs sham (16 sessions). For the 2018 trial by Haas et al. [56], means and standard deviations for the three experimental groups were combined, and compared to the single control group. The sensitivity analysis showed small effect sizes at short-term for headache intensity, frequency and disability (Figs. 10, 11, 12). Small effects were also found at long-term for headache intensity and frequency (Figs. 13, 14). The GRADE assessment [33] showed moderate quality of evidence for the sensitivity analysis results for each comparison. The GRADE evidence table for the sensitivity analysis is presented in Fig. 15.

## Discussion

The aim of this systematic review and meta-analysis was to assess the effects of manual and exercise therapy on headache intensity, frequency and other headache-related outcomes in patients experiencing CGHs.

Overall, this review found evidence consistently supporting the use of various manual therapy modalities for the management of CGH, based on nineteen RCTs, eight of which with a low RoB for the outcome measures of interest. In particular, there is stronger evidence favoring the use of spinal manipulation, spinal mobilization and Graston technique, while the positive effects of other interventions of interest are supported by fewer, low or unsure-RoB trials.

The meta-analysis of sham-controlled manual therapy trials showed moderate-to-large positive effects for manual therapy in reducing headache intensity, frequency and low-to-moderate positive effects on disability at short-term compared to sham. This meta-analysis also showed small-to-moderate and small positive effects for headache intensity and frequency at long-term. The GRADE assessment showed very low quality of evidence supporting manual therapy for the short-term estimates, and low quality of evidence of the long-term comparisons. A sensitivity meta-analysis including only low-RoB trials showed small effects of spinal manipulation for headache intensity and frequency at short and long-term, and for disability at short-term. The results of the GRADE assessment of the sensitivity meta-analysis showed moderate quality of evidence and can be interpreted as “the authors believe that the true effect is probably close to the estimated effect”. Considering the differences in the GRADE assessment and the resulting quality of evidence between the meta-analysis and the sensitivity analysis, the pooled estimates provide stronger evidence for the efficacy of spinal manipulation than other manual or exercise therapies. In particular, further studies are needed to allow data pooling and to assess the effectiveness of exercise therapy as a stand-alone treatment, but the integration with manual therapy appears to be effective based on relevant combinational trials included in this review [59, 63, 64, 66–69].

When comparing the results of this systematic review with a previous systematic review that only used conservative care as control [7], we notice that the trials pooled in this previous review were different and led to different results. The lack of effectiveness of spinal manipulation and mobilization reported by the previous systematic review compared to the moderate-size positive effects found in the current meta-analysis, strengthens the importance of comparing the interventions of interest to sham interventions. Another systematic review and meta-analysis [8] found a similar direction of results, although with generally smaller effect sizes for headache intensity, frequency and disability at both short and long term. The smaller effects seen in the [8] review are explained by a different grouping of trials (which included no-treatment comparators), and different treatment of individual trials [55, 56] in its meta-analysis.

Furthermore, the sensitivity analysis included in the present manuscript allows for a more robust interpretation of the effects of spinal manipulation, and provides higher-quality evidence.

Comparing the results of the present review to the clinical indications proposed by Cote et al. in previous guidelines [6], the existing recommendations for the use of manual therapy and exercise are strengthened, especially regarding spinal manipulation and mobilization. In fact, 10 of the 11 included trials of spinal manipulation and mobilization reported clinical and statistical superior effects for the experimental group compared to controls. Contrastingly, the evidence was limited to fewer trials with high or unsure risk of bias for other manual therapy interventions (myofascial trigger point therapy, dry needling, kinesio-taping, Graston technique, Dennerol cervical traction) and for exercise therapy. The guidelines’ manual therapy recommendations are strengthened further by the results of our meta-analysis, while meta-analysis was not feasible for exercise trials. Previous guidelines discourage combinations of manual therapy and low-load endurance cervico-scapular exercise, based on a single high-risk of bias trial [59]. The present systematic review found that the addition of Graston technique to an exercise plan provided statistical significant improvements compared to the exercise regime alone [67]. Consequently, although these findings are in line with existing guidelines, the evidence seems to suggest that clinicians could consider offering patients a mixed approach which combines manual therapy and stretching, isometric exercises and postural correction.

The Cote et al. guidelines [6] also provide indications on the dosage of such interventions, recommending a maximum of 10 manual therapy sessions. Nonetheless, one trial [56] included in our sensitivity meta-analysis reported a higher efficacy of spinal manipulation at 18 sessions, compared to 12 or 6 sessions. Consequently, although this systematic review confirms that spinal manipulation is the intervention with the greatest amount and quality of evidence available, a higher dose of interventions may be necessary to obtain statistically and clinically significant improvements, which contrasts with previous guidance.

Shared decision-making and patient education should be the basis of choosing an intervention, as per current literature and CGH guidelines [6]. To facilitate this process, the present review also considered MCIDs and adverse events wherever possible. MCIDs could be used to contextualize the review’s findings for three outcome measures (headache intensity with NPRS, headache frequency, disability measured by the NDI). To be meaningful to patients, changes in NPRS and NDI need to be at least 2.5 and 5.5 points [16], respectively, within four weeks; recognizing, however, that meaningfulness likely differs between groups of patients and that more research on context-sensitive MCIDs may be required. In the reviewed studies, MCIDs were largely reached, despite treatment intensities and dosages varying widely. Considering the context and time required to achieve the clinical benefits observed in the present review, the magnitude of the changes seems to justify the resources. Weighing intervention risks against patient-perceived benefits, it has been reported that up to 50% of patients receiving manual therapy can experience transient mild adverse effects. These are generally self-resolving within 48–72 h, which is lower than the risk with most drug therapies [13]. The incidence of adverse events reported in the included trials is well below 50%, and no serious adverse events were reported. While such data underlines the relative safety of manual therapy for CGH, patients should be informed about the possibility of experiencing transient adverse effects. Considering the results of this systematic review, the authors recommend that practitioners discuss with patients the available evidence regarding the effectiveness of manual and exercise therapy and alternative interventions as well as their costs and risks. This will promote realistic expectations for people experiencing CGH, supporting them to make an informed decision about their health.

To the authors’ knowledge, this is the first systematic review and meta-analysis of CGH trials to assess such a wide range of interventions and to analyze trials using different control interventions, which makes it the most comprehensive review available on CGH. Furthermore, the rigorous data pooling methodology, the presence of a sensitivity analysis based on low-RoB trials only, the thorough analysis of each trial and their MCIDs as well as the various GRADE assessments for each of the pooled estimates, allow a more specific interpretation of the findings, compared to previous systematic reviews and meta-analyses on this topic. Limitations to this review were the exclusion of trials in Chinese, the limited number of published trials, small group sizes, and the prevalence of trials with unclear or high RoB. Differences in trial design (notably choice of comparators and treatment dosage) limited the number of studies that could be pooled for meta-analysis. A notable challenge in trial design in the field of manual therapy and exercise therapy research is the intrinsic difficulty in patient and therapist blinding, and a limitation to this systematic review is that the included trials rarely evaluated the patient-blinding effectiveness. Consequently, even in sham-controlled trials it remains unclear whether the influence of patient expectations was adequately controlled [20–22]. Some of the included trials had further specific limitations. In both trials assessing trigger point therapy [51, 58], participants were included only when showing signs of a trigger point at the sternocleidomastoid muscle, which might not be representative of all people living with CGH and could limit the generalizability of these conclusions. Similarly, the presence of TMJ dysfunction as inclusion criterion in the trial by von Piekartz et al. [64] decreases the generalizability of the findings, although the results can be considered when making treatment recommendations specific to patients with TMJ dysfunction. Considering the concerns about methodological and reporting quality of the trial by Yang and Kang [65], it is the opinion of the authors of this systematic review that no conclusions should be drawn from this study.

Furthermore, only trials on spinal manipulation were included in the sensitivity meta-analysis, restricting the relevance of the meta-analysis to this particular intervention. Another common limitation in trials on physical therapy is that the standardized treatment procedures described in the intervention groups seldom reflect common practice, where the choice of the intervention is specific to the patient, rather than being standardized across patients. This can limit the translatability of guidelines to clinical practice [71]. A further limitation is that only 11 of the included trials were excluding participants with co-existing headaches, which could have similar characteristics to CGH and confound trial results. This and the considerable overlap across headache types in various diagnostic classifications, pose a considerable limitation to the systematic review. Nonetheless, it could be argued that due to the diagnostic challenges, this limitation might be considered inherent to headache trials [12]. In addition, 60% of the trials did not provide data on adverse events, which might keep readers unaware of possible major or minor complications experienced by participants. Considering the limitations described and the low-to-moderate quality of evidence found with GRADE, further *16 RCTs are expected and necessary to clarify the role of manual and exercise therapy, especially for interventions other than spinal manipulation. In order to generate more comparable and high-quality evidence for these interventions for CGH, future primary research on this topic should consider the limitations encountered in this systematic review.

## Conclusion

Manual therapy (with or without exercise therapy) appears to be a safe and effective intervention for CGH, and should be considered in the management of this condition, as already proposed by the latest guidelines [6]. The main body of evidence favours the use of spinal manipulation to reduce headache intensity, frequency and disability, but other forms of manual therapy and exercise therapy were found to be consistently beneficial for other outcomes across the trials. Future research with low-RoB RCTs, higher numbers of participants, better-defined headache populations, and more homogeneous trial designs is necessary to confirm these findings. The relevance for clinical practice is considerable, as reflected by the amount of clinical guidelines proposing some form of manual or physical therapy in the management of headaches, and the large number of patients seeking this type of intervention to manage their headache symptoms.

## Data Availability

The datasets used and/or analyzed during the current study are available from the corresponding author on reasonable request.

## Abbreviations

CGH: Cervicogenic headache
RCT: Randomized controlled trial
PRISMA: Preferred reporting items for systematic review and meta-analysis
PICO: Population intervention comparison outcome(s)
GRADE: Grading of recommendations, assessment, development and evaluations
RoB: Risk of bias
SMD: Standard mean difference
NPRS: Numeric pain rating scale
VAS: Visual analogue scale
NDI: Neck disability index
CROM: Cervical range of motion
CAS: Coloured analogue scale
MCID: Minimal clinically important difference
SNAG: Self-sustained naural apophyseal glide
TMJ: Temporo-mandibular joint

## References

[1] Al Khalili Y, Ly N, Murphy PB (2020). Cervicogenic headache.

[2] ICHD: Headache Classification Subcommitee of the International Headache Society (2018). The international classification of headache disorders. Cephalalgia.

[3] Sjaastad O, Fredriksen TA, Pfaffenrath V (1998). Cervicogenic headache: diagnostic criteria. Headache.

[4] Sjaastad O, Fredriksen T, Pareja JA (1999). Coexistence of cervicogenic headache and migraine without aura (?). Funct Neurol.

[5] Moore CS, Sibbritt DW, Adams J (2017). A critical review of manual therapy use for headache disorders: prevalence, profiles, motivations, communication and self-reported effectiveness. BMC Neurol.

[6] Cote P, Yu H, Shearer HM, Randhawa K, Wong JJ, Mior S, Ameis A, Carroll LJ, Nordin M, Varatharajan M, Sutton D, Southerst D, Jacobs C, Stupar M, Taylor-Vaisey A, Gross DP, Brison RJ, Paulden M, Ammendolia C, Cassidy JD, Loisel P, Marshall S, Bohay RN, Stapleton J, Lacerte M (2019). Non-pharmacological management of persistent headaches associated with neck pain: a clinical practice guideline from the Ontario protocol for traffic injury management (OPTIMa) collaboration. Eur J Pain.

[7] Coelho M, Ela N, Garvin A, Cox C, Sloan W, Palaima M, Cleland JA (2019). The effectiveness of manipulation and mobilization on pain and disability in individuals with cervicogenic and tension-type headaches: a systematic review and meta-analysis. Phys Ther Rev.

[8] Fernandez M, Moore C, Tan J, Lian D, Nguyen J, Bacon A, Christie B, Shen I, Waldie T, Simonet D, Bussieres A (2020). Spinal manipulation for the management of cervicogenic headache: a systematic review and meta-analysis. Eur J Pain.

[9] Freedland KE, King AC, Ambrosius WT, Mayo-Wilson E, Mohr DC, Czajkowski SM, Thabane L, Collins ML, Rebok GW, Treweek SP, Cook TD, Edinger JD, Stoney CM, Campo RA, Young-Hyman D, Riley WT (2019). The selection of comparators for randomized controlled trials of health-related behavioral interventions: recommendations of a NIH expert panel. J Clin Epidemiol.

[10] Page MJ, McKenzie JE, Bossuyt PM, Boutron I, Hoffman TC, Mulrow CD, Shamseer L, Tetzlaff JM, Akl EA, Brennan SE, Chou R, Glanville J, Grimshaw JM, Hrobjartsson A, Lalu MM, Li T, Loder EW, Mayo-Wilson E, McDonald S, McGuinness LA, Stewart LA, Thomas J, Tricco AC, Welch VA, Whiting P, Moher D (2021). The PRISMA 2020 statement: an upgraded guideline for reporting systematic reviews. BMJ.

[11] Santos CMC, Pimenta CAM, Nobre MRC (2007). The PICO strategy for the research question construction and evidence search. Rev Latino-Am Enfermagem.

[12] Luedtke K, Allers A, Schulte LH, May A (2016). Efficacy of interventions used by physiotherapists for patients with headache and migraine-systematic review and meta-analysis. Cephalalgia.

[13] Carnes D, Mars TS, Mullinger B, Froud R, Underwood M (2010). adverse events and manual therapy: a systematic review. Man Ther.

[14] Bielecki JE, Tadi P (2022). Therapeutic exercise.

[15] Luedtke K, Basener A, Bedei S, Castien R, Chaibi A, Falla D, Fernandez-de-las-Penas C, Gustaffson M, Hall T, Jull G, Kropp P, Madsen BK, Schefer B, Seng E, Steen C, Tuchin P, von Piekartz H, Wollesen B (2020). Outcome measures for assessing the effectiveness of non-pharmacological interventions in frequent episodic or chronic migraine: a Delphi study. BMJ Open.

[16] Young IA, Dunning J, Butts R, Cleland JA, Fernandez-de-las-Penas C (2019). Psychometric properties of the numeric pain rating scale and neck disability index in patients with cervicogenic headache. Cephalalgia.

[17] Covidence, https://www.covidence.org/; 2022 [accessed 10th-01–2022].

[18] McHugh ML (2012). Interrater reliability: the kappa statistic. Biomech Med.

[19] Furlan AD, Malmivaara A, Chou R, Maher CG, Deyo RA, Schoene M, Bronfort G, van Tulder MW (2015). 2015 updated method guideline for systematic reviews in the cochrane back and neck group. Spine.

[20] Hohenschurz-Schmidt D, Draper-Rodi J, Vase L, Scott W, McGregor A, Soliman N, MacMillan A, Olivier A, Cherian CA, Corcoran D, Abbey H, Freigang S, Chan J, Phalip J, Sørensen LN, Delafin M, Baptista M, Medforth N, Ruffini N, Andresen SS, Ytier S, Ali D, Hobday H, Santosa AA, Vollert J, Rice AS (2022). Blinding and sham control methods in trials of physical, psychological, and self-management interventions for pain (article I): a systematic review and description of methods. Pain.

[21] Hohenschurz-Schmidt D, Draper-Rodi J, Vase L, Scott W, McGregor A, Soliman N, MacMillan A, Olivier A, Cherian CA, Corcoran D, Abbey H, Freigang S, Chan J, Phalip J, Sørensen LN, Delafin M, Baptista M, Medforth N, Ruffini N, Andresen SS, Ytier S, Ali D, Hobday H, Santosa AA, Vollert J, Rice AS (2022). Blinding and sham control methods in trials of physical, psychological, and self-management interventions for pain (article II): a meta-analysis relating methods to trial results. Pain.

[22] Armijo-Olivo S, Fuentes J, da Costa BR, Saltaji H, Ha C, Cummings GG (2017). Blinding in physical therapy trials and its association with treatment effects. Am J Phys Med Rehabil.

[23] Viswanathan M, Ansari MT, Berkman ND, Chang S, Hartling L, McPheeters M, Santaguida L, Shamliyan T, Singh K, Tsertsvadze A, Treadwell JR. Assessing the risk of bias of individual studies in systematic reviews of health care interventions. 2012. In: Methods guide for effectiveness and comparative effectiveness reviews [Internet]. Rockville (MD): Agency for Healthcare Research and Quality (US); 2008-. Available from: https://www.ncbi.nlm.nih.gov/books/NBK91433/ (Accessed 1st July 2022) 22479713

[24] Salas Apasa JA, Ariel Franco JV, Meza N, Madrid E, Loezar C, Garegnani L (2021). Minimal clinically importance difference: the basics. Medwave.

[25] Hawker GA, Mian S, Kendzerska T, French M (2011). Measures of adult pain: visual analog scale for pain (VAS pain), numeric rating scale for pain (NRS Pain), McGill pain questionnaire (MPQ), short-form McGill pain questionnaire (SF-MPQ), chronic pain grade scale (CPGS), short form-36 bodily pain scale (SF-36 BPS), and measure of intermittent and constant osteoarthritis pain (ICOAP). Arthritis Care Res.

[26] Fleming R, Forsythe S, Cook C (2007). Influential variables associated with outcomes in patients with cervicogenic headache. J Man Manip Ther.

[27] McGrath P, Seifert CE, Speechley KN, Booth JC, Stitt L, Gibson MC (1996). A new analogue scale for assessing children’s pain: an initial validation study. Pain.

[28] Underwood MR, Barnett AG, Vickers MR (1999). Evaluation of two time-specific back pain outcome measures. Spine.

[29] Jull GA, Stanton WR (2004). Predictors of responsiveness to physiotherapy management of cervicogenic headache. Cephalalgia.

[30] Review Manager (RevMan) [Computer program]. Version 5.4. The Cochrane Collaboration, 2020

[31] Cohen J (1988). Statistical power analysis for the behavioral sciences.

[32] Bronfort G, Nilsson N, Haas M, Evans RL, Goldsmith CH, Assendelft WJJ, Buter LM (2004). Non-invasive physical treatments for chronic/recurrent headache. Cochrane Database Syst Rev.

[33] Guyatt G, Oxman AD, Akl EA, Kunz R, Vist G, Brozek J, Norris S, Falck-Ytter Y, Glasziou P, DeBeer H, Jeaschke R, Rind D, Meerophl J, Dahm P, Schunemann HJ (2011). GRADE guidelines: 1. Introduction-GRADE evidence profiles and summary of findings tables. J Clin Epidemiol.

[34] Guyatt G, Oxman AD, Vist G, Kunz R, Brozek J, Alonso-Coello P, Montori V, Akl AE, Djulbegovic B, Falck-Ytter Y, Norris SL, Williams JW, Atkins D, Meerpohl J, Schunemann HJ (2011). GRADE guidelines 4: rating the quality of evidence- study limitations (risk of bias). J Clin Epidemiol.

[35] Guyatt G, Oxman AD, Kunz R, Woodcock J, Brozek J, Helfand M, Alonso-Coello P, Glasziou P, Jaeschke R, Akl AE, Norris SL, Vist G, Dahm P, Shukla VK, Higgins J, Falck-Ytter Y, Schunemann HJ (2011). GRADE guidelines 7: rating the quality of evidence - inconsistency. J Clin Epidemiol.

[36] Guyatt G, Oxman AD, Kunz R, Brozek J, Alonso-Coello P, Rind D, Devereaux PJ, Montori VM, Bo F, Vist G, Jaeschke R, Williams JW, Murad MH, Sinclair D, Falck-Ytter Y, Meerpohl J, Whittington C, Thorlund K, Andrews J, Schunemann HJ (2011). GRADE guidelines 6: rating the quality of evidence - imprecision. J Clin Epidemiol.

[37] Guyatt G, Oxman AD, Kunz R, Woddocock J, Brozek J, Helfand M, Alonso-Coello P, Falck-Ytter Y, Jaeschke R, Vist G, Akl AE, Post PN, Norris N, Meerpohl J, Shukla VK, Nasser M, Schunemann HJ (2011). GRADE guidelines 8: rating the quality of evidence - indirectness. J Clin Epidemiol.

[38] Higgins JPT, Thomas J, Chandler J, Cumpston M, Li T, Page MJ, Welch VA (2019). Cochrane handbook for systematic reviews of interventions.

[39] Guyatt G, Oxman AD, Vist G, Kunz R, Falck-Ytter Y, Alonso-Coello P, Schunemann HJ (2008). GRADE: an emerging consensus on rating quality of evidence and strength of recommendations. BMJ.

[40] Langevin P, Fait P, Frémont P, Roy JS (2019). Cervicovestibular rehabilitation in adult with mild traumatic brain injury: a randomised controlled trial protocol. BMC Sports Sci Med Rehabil.

[41] Svedmark A, Djupsjöbacka M, Häger C, Jull G, Björklund M (2016). Is Tailored treatment superior to non-tailored treatment for pain and disability in women with non-specific neck pain? a randomized controlled trial. BMC Musculoskelet Disord.

[42] Vernon H, Borody C, Harris G, Muir B, Goldin J, Dinulos M (2015). A randomized pragmatic clinical trial of chiropractic care for headaches with and without a self-acupressure pillow. J Manip Physiol Ther.

[43] Daher A, Carel RS, Tzipi K, Esther H, Dar G (2020). The effectiveness of an aerobic exercise training on patients with neck pain during a short- and long-term follow-up: a prospective double-blind randomized controlled trial. Clin Rehabil.

[44] Ylinen J, Nikander R, Nykänen M, Kautiainen H, Häkkinen A (2010). Effect of neck exercises on cervicogenic headache: a randomized controlled trial. J Rehabil Med.

[45] Whittingham W, Nilsson N (2001). Active range of motion in the cervical spine increases after spinal manipulation (toggle recoil). J Manipulative Physiol Ther.

[46] Ramezani E, Arab AM (2017). The effect of suboccipital myofascial release technique on cervical muscle strength of patients with cervicogenic headache. PTJ..

[47] von Piekartz H, Hall T (2013). Orofacial manual therapy improves cervical movement impairment associated with headache and features of temporomandibular dysfunction: a randomized controlled trial. Man Ther.

[48] Haas M, Aickin M, Vavrek D (2010). A preliminary path analysis of expectancy and patient-provider encounter in an open-label randomized controlled trial of spinal manipulation for cervicogenic headache. J Manip Physiol Ther.

[49] Borusiak P, Biedermann H, Bosserhoff S, Opp J (2010). Lack of efficacy of manual therapy in children and adolescents with suspected cervicogenic headache: results of a prospective, randomized, placebo-controlled, and blinded trial. Headache.

[50] Khan M, Shahzad S, Soomro RR (2014). Efficacy of C1–C2 sustained natural apophyseal glide (SNAG) versus posterior anterior vertebral mobilization (PAVMs) in the management of cervicogenic headache. J Basic Appl Sci.

[51] Bodes-Pardo G, Pecos-Martin D, Gallego-Izquierdo T, Salom-Moreno J, Fernandez-de-las-Penas C, Ortega-Santiago R (2013). Manual treatment for cervicogenic headache and active trigger points in the sternocleidomastoid muscle: a pilot randomized clinical trial. J Manipulative Physiol Ther.

[52] Chaibi A, Knackstedt H, Tuchin PJ, Russell MB (2017). Chiropractic spinal manipulative therapy for cervicogenic headache: a single-blinded, placebo, randomized controlled trial. BMC Res Notes.

[53] Dunning JR, Butts R, Mourad F, Young I, Fernandez-de-las-Penas C, Hagins M, Stanislawski T, Donley J, Buck D, Hooks TR, Cleland JA (2016). Upper cervical and upper thoracic manipulation versus mobilization and exercise in patients with cervicogenic headache: a multi-center randomized clinical trial. BMC Musculoskelet Disord.

[54] Esin OR, Khairullin IK, Esin RG (2018). Efficiency of kinesio taping in adolescents with cervicogenic headache: a blind placebo-controlled study. BioNanoScience.

[55] Haas M, Spegman A, Peterson D, Aickin M, Vavrek D (2010). Dose response and efficacy of spinal manipulation for chronic cervicogenic headache: a pilot randomized controlled trial. Spine J.

[56] Haas M, Bronfort G, Evans R, Schulz C, Vavrek D, Takaki L, Hanson L, Leininger B, Neradilek MB (2018). Dose-response and efficacy of spinal manipulations for care of cervicogenic headache: a dual-center randomized controlled trial. Spine J.

[57] Hall T, Chan HT, Christensen L, Odenthal B, Wells C, Robinson K (2007). Efficacy of a C1–C2 Self-sustained natural apophyseal glide (SNAG) in the management of cervicogenic headache. J Orthop Sports Phys Ther.

[58] Jafari M, Bahrpeyma F, Togha M (2017). Effect of ischemic compression for cervicogenic headache and elastic behavior of active trigger point in the sternocleidomastoid muscle using ultrasound imaging. J Bodyw Mov Ther.

[59] Jull G, Trott P, Potter H, Zito G, Niere K, Shirley D, Emberson J, Marschner I, Richardson C (2002). A randomized controlled trial of exercise and manipulative therapy for cervicogenic headache. Spine.

[60] Malo-Urriès M, Tricas-Moreno JM, Estebanez-de-Miguel E, Hidalgo-Garcia C, Carrasco-Uribarren A, Cabanillas-Barea S (2017). Immediate effects of upper cervical translatoric mobilization on cervical mobility and pressure pain threshold in patients with cervicogenic headache: a randomized controlled trial. J Manip Physiol Ther.

[61] Nilsson N, Christensen HW, Hartvigsen J (1997). The effect of spinal manipulation in the treatment of cervicogenic headache. J Manip Physiol Ther.

[62] Sedighi A, Ansari NN, Naghdi S (2017). Comparison of acute effects of superficial and deep dry needling into trigger points of suboccipital and upper trapezius muscles in patients with cervicogenic headache. J Bodyw Mov Ther.

[63] Sharma A, Hameed UA, Grover S (2011). Multimodal therapy in cervicogenic headache- a randomized controlled trial. Indian J Physiother Occup Ther.

[64] Von Piekartz H, Luedtke K (2011). Effect of treatment of temporomandibular disorders (TMD) in patients with cervicogenic headache: single-blind, randomized controlled study. Cranio J Craniomandib Pract.

[65] Yang DJ, Kang DH (2017). Comparison of muscular fatigue and tone of neck according to craniocervical flexion exercise and suboccipital relaxation in cervicogenic headache patients. J Phys Ther Sci.

[66] Youssef EF, Shanb AA (2013). Mobilization versus massage therapy in the treatment of cervicogenic headache: a clinical study. J Back Musculoskelet Rehabil.

[67] Abdel-Aal NB, Elsayyad MM, Megahed AA (2021). Short-term effect of adding Graston technique to exercise program in treatment of patients with cervicogenic headache: a single-blinded, randomized controlled trial. Eur J Phys Rehabil Med.

[68] Dunning J, Butts R, Zacharko N, Fandry K, Young I, Wheeler K, Fernandez-de-las-Penas C (2021). Spinal manipulation and perineural electrical dry needling in patients with cervicogenic headache: a multicenter randomized clinical trial. Spine J.

[69] Lerner-Lentz A, O’Halloran B, Donaldson M, Cleland JA (2021). Pragmatic application of manipulation versus mobilization to the upper segments of the cervical spine plus exercise for treatment of cervicogenic headache: a randomized clinical trial. J Man Manip Ther.

[70] Moustafa IM, Diab A, Shousha T, Harrison DE (2021). Does restoration of sagittal cervical alignment improve cervicogenic headache pain and disability: a 2-year pilot randomized controlled trial. Heliyon.

[71] Vander Schaaf EB, Seashore CJ, Randolph GD (2015). Translating clinical guidelines into practice: challenges and opportunities in a dynamic health care environment. NCMJ.

